# Supplementary figures and images for: Comparative transcriptome analysis provides new insights into the response of common bean to infection by race 65 of Colletotrichum lindemuthianum
Source: PLoS One. 2025 Oct 17;20(10):e0314188. doi: 10.1371/journal.pone.0314188 (PMC12533873; doi:10.1371/journal.pone.0314188)

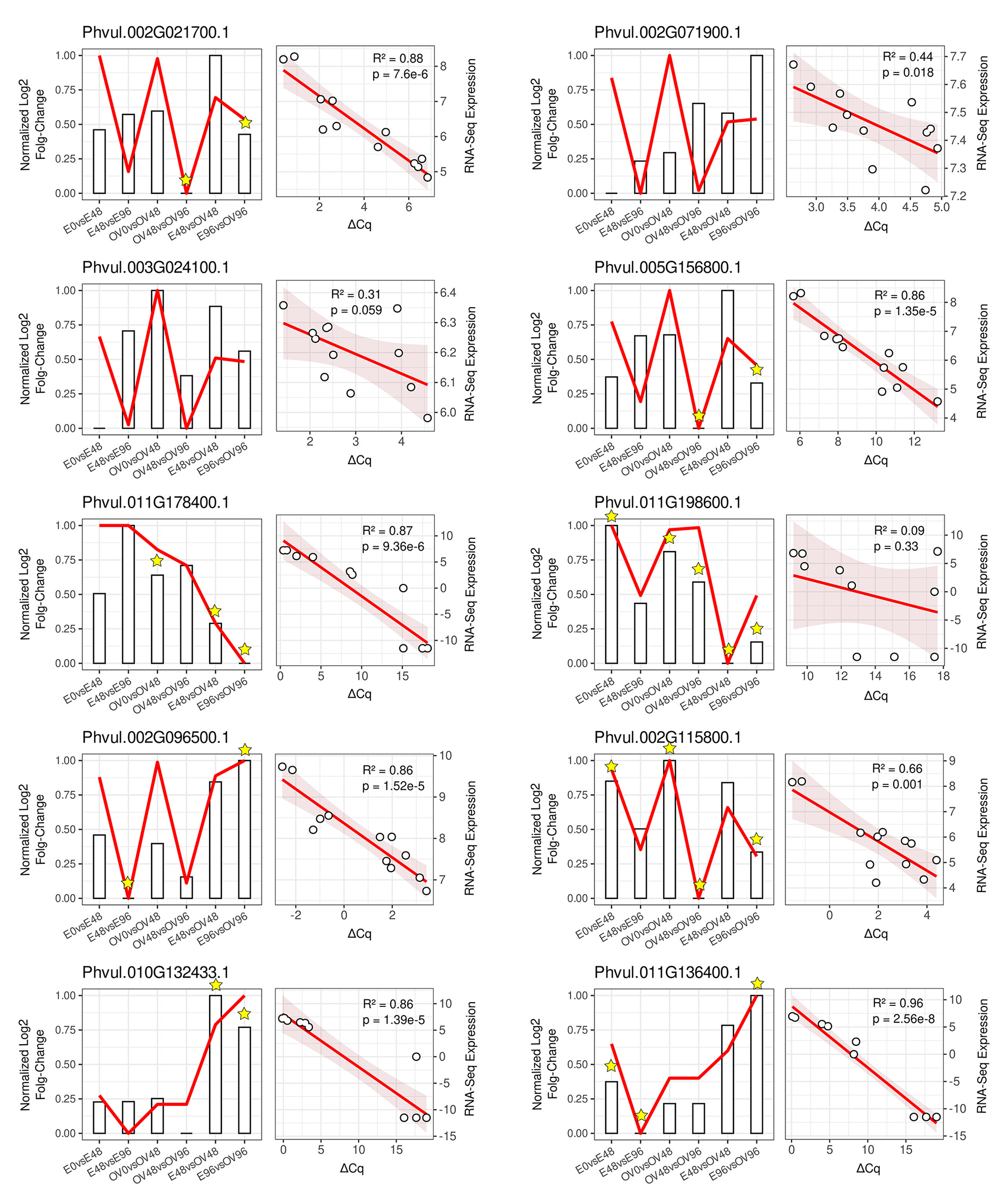

Supplement: S1 Fig — (TIF) [file pone.0314188.s001.tif]
