# Supplementary material for: Comparative transcriptome analysis provides new insights into the response of common bean to infection by race 65 of Colletotrichum lindemuthianum
Source: PLoS One. 2025 Oct 17;20(10):e0314188. doi: 10.1371/journal.pone.0314188 (PMC12533873; doi:10.1371/journal.pone.0314188)
Supplement: Attachment — Submitted filename: PONE-D-24-50843.pdf [file pone.0314188.s016.pdf]

PLOS ONE

Comparative transcriptome analysis provides new insights into the response of common bean to infection by race 65 of Colletotrichum lindemuthianum

--Manuscript Draft--

**Manuscript Number:**	PONE-D-24-50843
**Article Type:**	Research Article
**Full Title:**	Comparative transcriptome analysis provides new insights into the response of common bean to infection by race 65 of Colletotrichum lindemuthianum
**Short Title:**	Transcriptome Insights into Common Bean Response to Colletotrichum lindemuthianum Race 65
**Corresponding Author:**	Welison Andrade Pereira, Ph.D.<br>Federal University of Lavras: Universidade Federal de Lavras<br>Lavras, Minas Gerais BRAZIL
**Keywords:**	Phaseolus vulgaris; Colletotrichum lindemuthianum; Plant-pathogen interaction; Anthracnose; RNA-Seq; Gene coexpression network
**Abstract:**	The farming of common beans is essential for the human population, due to its nutritional value and cultural, economic, and social perspectives. Enhancing productivity is a strategic objective of common bean breeding, yet the crop is negatively impacted by biotic and abiotic stresses. Anthracnose, caused by Colletotrichum lindemuthianum, is a major challenge for beans, particularly in tropical countries such as Brazil, where pathogen diversity is vast. Race 65 of C. lindemuthianum is notable due to its widespread occurrence and high genetic and virulence diversity. Understanding the molecular bases of resistance and susceptibility to specific pathogen races can accelerate the development of superior cultivars. Despite its significance, no global gene expression studies have addressed the interactions between bean genotypes and race 65. In this study, a comprehensive transcriptome analysis was performed on two Brazilian cultivars, one resistant and one susceptible to race 65. Total RNA was extracted from leaves at 0, 48, and 96 hours after inoculation and sequenced using the Illumina NextSeq 500 platform. By analyzing contrasts over the infection time course, we identified several candidate genes associated with resistance and susceptibility mechanisms. Resistance-related genes included those involved in pathogen recognition, such as kinases and NB-LRR (nucleotide-binding and leucine-rich repeat) genes, as well as genes involved in the phenylpropanoid, glycerolipid biosynthesis, and linoleic acid pathways. Additionally, genes related to pathogenesis-related proteins (PR) and cell wall remodeling were triggered upon pathogen recognition in the resistant genotype. In the susceptible genotype, auxin-mediated signaling and sugar transport genes were prominent. Gene coexpression network analysis revealed a significant correlation among resistance-associated genes, forming a robust network. Our findings shed light on the molecular-genetic mechanisms underlying the response of common beans to race 65 of this pathogen.
**Order of Authors:**	Caroline Marcela da Silva Dambroz
	Alexandre Hild Aono, PhD
	Larissa Carvalho Costa, PhD
	Evandro Novaes, PhD
	Welison Andrade Pereira, Ph.D.
**Additional Information:**	
**Question**	**Response**
**Financial Disclosure**<br><br>Enter a financial disclosure statement that describes the sources of funding for the	Yes

Powered by Editorial Manager® and ProduXion Manager® from Aries Systems Corporation

work included in this submission. Review the submission guidelines for detailed requirements. View published research articles from *PLOS ONE* for specific examples.<br><br>This statement is required for submission and **will appear in the published article** if the submission is accepted. Please make sure it is accurate.<br><br>**Funded studies**<br>Enter a statement with the following details:<br>• Initials of the authors who received each award<br>• Grant numbers awarded to each author<br>• The full name of each funder<br>• URL of each funder website<br>• Did the sponsors or funders play any role in the study design, data collection and analysis, decision to publish, or preparation of the manuscript?<br><br>Did you receive funding for this work?	
Please add funding details.<br>as follow-up to "**Financial Disclosure**<br><br>Enter a financial disclosure statement that describes the sources of funding for the work included in this submission. Review the submission guidelines for detailed requirements. View published research articles from *PLOS ONE* for specific examples.<br><br>This statement is required for submission and **will appear in the published article** if the submission is accepted. Please make sure it is accurate.	During the course of this study, CMSD received a scholarship from the Conselho Nacional do Desenvolvimento Científico e Tecnológico (CNPq).

Powered by Editorial Manager® and ProduXion Manager® from Aries Systems Corporation

**Funded studies**<br>Enter a statement with the following details:<br>• Initials of the authors who received each award<br>• Grant numbers awarded to each author<br>• The full name of each funder<br>• URL of each funder website<br>• Did the sponsors or funders play any role in the study design, data collection and analysis, decision to publish, or preparation of the manuscript?<br><br>Did you receive funding for this work?"	
Please select the country of your main research funder (please select carefully as in some cases this is used in fee calculation).<br>as follow-up to "**Financial Disclosure**<br><br>Enter a financial disclosure statement that describes the sources of funding for the work included in this submission. Review the submission guidelines for detailed requirements. View published research articles from *PLOS ONE* for specific examples.<br><br>This statement is required for submission and **will appear in the published article** if the submission is accepted. Please make sure it is accurate.<br><br>**Funded studies**<br>Enter a statement with the following details:<br>• Initials of the authors who received each award<br>• Grant numbers awarded to each author<br>• The full name of each funder<br>• URL of each funder website<br>• Did the sponsors or funders play any role in the study design, data collection and analysis, decision to publish, or preparation of the manuscript?<br><br>Did you receive funding for this work?"	BRAZIL - BR
**Competing Interests**	The authors have declared that no competing interests exist.

Use the instructions below to enter a competing interest statement for this submission. On behalf of all authors, disclose any competing interests that could be perceived to bias this work—acknowledging all financial support and any other relevant financial or non-financial competing interests.<br><br>This statement is required for submission and **will appear in the published article** if the submission is accepted. Please make sure it is accurate and that any funding sources listed in your Funding Information later in the submission form are also declared in your Financial Disclosure statement.<br><br>View published research articles from *PLOS ONE* for specific examples.<br><br>**NO authors have competing interests**<br><br>Enter: *The authors have declared that no competing interests exist.*<br><br>**Authors with competing interests**<br><br>Enter competing interest details beginning with this statement:<br><br>*I have read the journal's policy and the authors of this manuscript have the following competing interests: [insert competing interests here]*<br><br>* typeset	
**Ethics Statement**<br><br>Enter an ethics statement for this submission. This statement is required if the study involved:<br><br>• Human participants<br>• Human specimens or tissue<br>• Vertebrate animals or cephalopods<br>• Vertebrate embryos or tissues	N/A

Powered by Editorial Manager® and ProduXion Manager® from Aries Systems Corporation

• Field research<br><br>Write "N/A" if the submission does not require an ethics statement.<br><br>General guidance is provided below. Consult the submission guidelines for detailed instructions. **Make sure that all information entered here is included in the Methods section of the manuscript.**	

Powered by Editorial Manager® and ProduXion Manager® from Aries Systems Corporation

**Format for specific study types**<br><br>**Human Subject Research (involving human participants and/or tissue)**<br>• Give the name of the institutional review board or ethics committee that approved the study<br>• Include the approval number and/or a statement indicating approval of this research<br>• Indicate the form of consent obtained (written/oral) or the reason that consent was not obtained (e.g. the data were analyzed anonymously)<br><br>**Animal Research (involving vertebrate animals, embryos or tissues)**<br>• Provide the name of the Institutional Animal Care and Use Committee (IACUC) or other relevant ethics board that reviewed the study protocol, and indicate whether they approved this research or granted a formal waiver of ethical approval<br>• Include an approval number if one was obtained<br>• If the study involved *non-human primates*, add *additional details* about animal welfare and steps taken to ameliorate suffering<br>• If anesthesia, euthanasia, or any kind of animal sacrifice is part of the study, include briefly which substances and/or methods were applied<br><br>**Field Research**<br><br>Include the following details if this study involves the collection of plant, animal, or other materials from a natural setting:<br>• Field permit number<br>• Name of the institution or relevant body that granted permission	
**Data Availability**<br><br>Authors are required to make all data underlying the findings described fully available, without restriction, and from the time of publication. PLOS allows rare exceptions to address legal and ethical concerns. See the PLOS Data Policy and FAQ for detailed information.	Yes - all data are fully available without restriction

A Data Availability Statement describing where the data can be found is required at submission. Your answers to this question constitute the Data Availability Statement and **will be published in the article**, if accepted.<br><br>**Important:** Stating 'data available on request from the author' is not sufficient. If your data are only available upon request, select 'No' for the first question and explain your exceptional situation in the text box.<br><br>Do the authors confirm that all data underlying the findings described in their manuscript are fully available without restriction?	
**Describe where the data may be found in full sentences. If you are copying our sample text, replace any instances of XXX with the appropriate details.**<br><br>• If the data are **held or will be held in a public repository**, include URLs, accession numbers or DOIs. If this information will only be available after acceptance, indicate this by ticking the box below. For example: *All XXX files are available from the XXX database (accession number(s) XXX, XXX.*).<br>• If the data are all contained **within the manuscript and/or Supporting Information files**, enter the following: *All relevant data are within the manuscript and its Supporting Information files.*<br>• If neither of these applies but you are able to provide **details of access elsewhere**, with or without limitations, please do so. For example:<br><br>*Data cannot be shared publicly because of [XXX]. Data are available from the XXX Institutional Data Access / Ethics Committee (contact via XXX) for researchers who meet the criteria for access to confidential data.*<br><br>*The data underlying the results presented in the study are available from (include the name of the third party*	We are currently conducting a series of analyses that, due to their involvement in multiple projects and the need for originality, cannot be made publicly available at this time. However, we are committed to sharing these analyses with the public as soon as we have completed further evaluations of the same data.

and contact information or URL).<br>• This text is appropriate if the data are owned by a third party and authors do not have permission to share the data.<br><br>* typeset	
Additional data availability information:	Tick here if the URLs/accession numbers/DOIs will be available only after acceptance of the manuscript for publication so that we can ensure their inclusion before publication.; Tick here if your circumstances are not covered by the questions above and you need the journal's help to make your data available.

Manuscript Click here to access/download;Manuscript;Phavu-Collet_RNA-Seq_05112024.docx

Comparative transcriptome analysis provides new insights into the response of common bean to infection by race 65 of *Colletotrichum lindemuthianum*

Caroline Marcela da Silva Dambroz[1¶], Alexandre Hild Aono[2¶], Larissa Carvalho Costa[3¶], Evandro Novaes[1], Welison Andrade Pereira[1*]

[1] Department of Biology, Federal University of Lavras, Lavras, Minas Gerais, Brazil

[2] Molecular Biology and Genetic Engineering Center (CBMEG), University of Campinas (UNICAMP), Campinas, SP, Brazil

[3] Plant Germplasm Quarantine Program, Animal and Plant Health Inspection Service, United States Department of Agriculture, Beltsville, MD, United States.

[*] Corresponding author

E-mail: welison.pereira@ufla.br

[¶] These authors contributed equally to this work.

Abstract

The farming of common beans is essential for the human population, due to its nutritional value and cultural, economic, and social perspectives. Enhancing productivity is a strategic objective of common bean breeding, yet the crop is negatively impacted by biotic and abiotic stresses. Anthracnose, caused by *Colletotrichum lindemuthianum*, is a major challenge for beans, particularly in tropical countries such as Brazil, where pathogen diversity is vast. Race 65 of *C. lindemuthianum* is notable due to its widespread occurrence and high genetic and virulence diversity. Understanding the molecular bases of resistance and susceptibility to specific pathogen races can accelerate the development of superior cultivars. Despite its significance, no global gene expression studies have addressed the interactions between bean genotypes and race 65. In this study, a comprehensive transcriptome analysis was performed on two Brazilian cultivars, one resistant and one susceptible to race 65. Total RNA was extracted from leaves at 0, 48, and 96 hours after inoculation and sequenced using the Illumina NextSeq 500 platform. By analyzing contrasts over the infection time course, we identified several candidate genes associated with resistance and susceptibility mechanisms. Resistance-related genes included those involved in pathogen recognition, such as kinases and NB-LRR (nucleotide-binding and leucine-rich repeat) genes, as well as genes involved in the phenylpropanoid, glycerolipid biosynthesis, and linoleic acid pathways. Additionally, genes related to pathogenesis-related proteins (PR) and cell wall remodeling were triggered upon pathogen recognition in the resistant genotype. In the susceptible genotype, auxin-mediated signaling and sugar transport genes were prominent. Gene coexpression network analysis revealed a significant correlation among resistance-associated genes, forming a robust network. Our findings shed light on the molecular-

genetic mechanisms underlying the response of common beans to race 65 of this pathogen.

Introduction

Anthracnose, caused by the hemibiotrophic fungus *Colletotrichum lindemuthianum* (Sacc & Magn) Scribn, is one of the most economically significant diseases affecting common beans [1]. This disease is particularly prevalent in tropical and subtropical regions where climatic conditions favor pathogen development [2]. It impacts the quality of grains and pods and can severely reduce crop yields. Integrated disease management strategies include cultural practices, chemical control, and the use of resistant cultivars, with genetic resistance being considered the most efficient and safe method [3]. However, developing common bean cultivars with durable resistance to this pathogen is challenging, mainly due to the high virulence diversity of *C. lindemuthianum* [2].

The resistance of common beans to *C. lindemuthianum* has been characterized as predominantly qualitative, following the gene-to-gene model [4]. Approximately 23 loci with independent effects have been identified in different common bean varieties [5]. Despite the predominance of major resistance genes, quantitative trait loci (QTLs) conferring partial resistance to different races of *C. lindemuthianum* have also been identified [6–8]. Genetic mapping has revealed that these resistance loci are organized into complex clusters, comprising closely linked genes that confer resistance to specific races of the pathogen [3,7,9,10].

For some time now, race 65 of *C. lindemuthianum* has been one of the most prevalent in bean crops in Brazil [11] and one of the reasons for this persistence can be explained by its high genetic diversity and virulence, which come being identified among

its isolates [12–14]. In beans, in turn, seven major loci (*Co*-01, *Co*-02, *Co*-03, *Co*-04, *Co*-05, *Co*-06 and *Co*-15) were identified on different chromosomes conferring resistance to this race and its isolates [14–18]. However, recent studies demonstrate the specificity of resistance genes for different race 65 isolates [14]. Although knowledge of loci associated with common bean resistance to different races of *C. lindemuthianum* has advanced, a comprehensive understanding of the genes within these loci, their functional mechanisms, and interactions in host defense pathways remains crucial. This knowledge is essential for developing more effective strategies to obtain common bean cultivars with durable resistance to this pathogen [19].

Proteins encoded by resistance-related genes can act at various stages of the defense process, including pathogen recognition, triggering secondary defense responses, and participating in metabolic pathways [20]. For example, genes encoding proteins with kinase domains can function as pattern recognition receptors (PRRs). These receptors recognize pathogen-associated molecular patterns (PAMPs) and activate the defense response [21]. Within the superfamily of protein kinase-encoding genes (kinome), several leucine-rich repeat receptor-like protein kinases (LRR-RLK) have been associated with anthracnose resistance loci [18,21,22]. Other genes identified within resistance loci encode proteins with nucleotide-binding domains and leucine-rich repeats (NBS-LRR) [18]. These proteins can recognize pathogen effectors through protein-protein interactions, monitor the presence and function of other important host proteins, and induce effector-triggered immunity (ETI) upon detecting any alterations [23]. In all cases, the activation of the defense response initiates a signaling cascade, with proteins involved in different metabolic pathways triggering a series of responses that culminate in successful plant defense [20].

To increase the level of clarification, several studies have been carried out on the differential expression of candidate genes in specific bean cultivars resistant and susceptible to different races of *C. lindemuthianum* [24–27]. Regarding race 65, Silva et al. (2021) observed elevated expression levels of genes associated with pathogenesis and receptor kinases in a resistant bean cultivar using RT-qPCR. Padder et al. (2016) highlighted differences concerning the biotrophic and necrotrophic phases of the disease, along with specific genes located close to the Co-1 locus. Transcriptome analyses conducted on common bean lines resistant to races 73 [29], 100, and C531 [30] highlighted the importance of defense pathways mediated by the hormones ethylene, jasmonic acid, and salicylic acid. Alvarez-Diaz et al. (2022) suggested that the differences between compatible and incompatible interactions might be more related to the timing and intensity of gene expression rather than to the repertoire of differentially expressed genes (DEGs).

Given the specificity of genetic control in response to different races of *C. lindemuthianum*, coupled with the importance of race 65 for the crop, our study aimed to explore the transcriptional profiles of two common bean cultivars–resistant and susceptible to race 65 across an infection time course following artificial inoculation. Our findings offer valuable insights into the molecular mechanisms involved in the response of *Phaseolus vulgaris* to this specific isolate of race 65, thereby enhancing our comprehension of the molecular-genetic mechanisms involved in both compatible and incompatible interactions between *P. vulgaris* and *C. lindemuthianum*.

Material and methods

Plant material

The common bean cultivars BRS Estilo and Ouro Vermelho were selected for this study. Both are Brazilian common bean cultivars of Mesoamerican origin, commercially available to farmers. According to [14], these cultivars differ at a single locus in their reaction to race 65 of *C. lindemuthianum* (Isolate Lv134). Ouro Vermelho carries the dominant alleles responsible for resistance, while BRS Estilo carries the recessive alleles, conferring susceptibility.

To confirm the contrast reactions of BRS Estilo and Ouro Vermelho cultivars to isolate Lv134, race 65, their seeds were sown in polyethylene trays with individual cells filled with Plantmax substrate. The trial was conducted in a greenhouse under optimal conditions for humidity and temperature (relative humidity - RH of 95% and 24°C) to support germination and seedling development. After the primary leaves had expanded, the plants were inoculated with this fungal isolate to assess symptoms. The experiment followed a completely randomized design, in a 2 (cultivars) x 3 (collection times) factorial scheme, with three replicates per treatment.

Preparation of the conidia suspension and inoculation with *C. lindemuthianum* race 65

The isolate Lv 134, race 65 of *C. lindemuthianum* belongs to the fungal collection of the Plant Disease Resistance Laboratory at the Universidade Federal de Lavras (Brazil). Small mycelium fragments from this isolate, cultured on M3 medium plates [31], were transferred onto sterilized bean pods placed in test tubes containing a solidified water agar

base (3.75 g of agar to 250 ml of water) [32]. To optimize fungal sporulation, inoculated pods were incubated in the dark at 22ºC for 15 days.

Inoculum preparation involved adding distilled water to the tubes and scraping conidia from pod surfaces using a platinum loop. The conidia suspension was filtered through gauze to remove the mycelial fragments and adjusted to a standardized concentration of 1.2 x 10^6 conidia/ml using a Neubauer chamber under a microscope [33].

The conidia suspension was inoculated by spraying both sides of leaves and stems until runoff [34]. Inoculation was carried out after the primary leaves had fully opened. Plants were placed in a mist chamber (RH 100% and 25±2°C) for 48 hours, and then transferred to a greenhouse (RH 95% and 24°C) for an additional seven days before symptom assessment. As an experimental control, a group of plants from both common bean varieties used in this study were inoculated with water.

Collection of leaf material and assessment of symptoms

Leaf samples were collected at 0-, 48-, and 96-hours post-inoculation (hpi). Two halves of primary leaves from each plant were placed in 2.0 mL microtubes and immediately flash-frozen in liquid nitrogen for preservation. The samples were then stored at -80°C for later RNA extraction, while the remaining halves of the primary leaves were left on the plants for symptom assessment.

Each plant was individually evaluated nine days after inoculation using a descriptive scale from 1 to 9 [35]. Plants with a score equal to or below three were classified as resistant, while those scoring above three were considered susceptible. Statistical analysis was conducted using Student's t-test at a 5% significance level in R software [36].

RNA extraction, cDNA library construction, and sequencing

Total RNA was extracted from 12 samples, corresponding to the two common bean cultivars selected (BRS Estilo and Ouro Vermelho), collected at three time points (0, 48, and 96 hai), with two biological replicates for each treatment. RNA isolation was performed using the RNATRIzol® Isolation Reagent (Invitrogen) following the manufacturer's recommendations, with slight adjustments as described by [37]. RNA extracts were subsequently treated using TURBOTM DNAse (ThermoFisher) to remove any residual DNA, according to the manufacturer's instructions. The RNA concentration of each sample was quantified using Qubit (Qiagen), and RNA integrity number (RIN) was assessed using the Agilent 4200 TapeStation system.

RNA samples from each accession were diluted to a final concentration of 100ng/µl. Before library preparation, mRNA was enriched using the NEBNext® Poly(A) mRNA Magnetic Isolation Module (New England BioLabs). RNA-Seq libraries were then prepared using the TruSeq Stranded Total RNA Library Plant Kit (Illumina, Inc.) according to the manufacturer's instructions. After quantification using the TapeStation 4200 (Agilent), each library was normalized and pooled in equimolar ratios for sequencing on the Illumina NextSeq 500 platform (Illumina, Inc.) at the USDA-APHIS PGQP laboratory, employing a 75-cycle high-output sequencing kit (2 x 75 bp, paired-end).

Bioinformatics analysis

The quality of the sequencing reads was assessed using FastQC v.0.11.7 [38]. Adapter sequences and bases with a Q-Score below 15 in a four-base window were removed using Trimmomatic v.0.39 [39], and reads shorter than 32 bases were discarded. The filtered

reads were aligned to the reference genome of *P. vulgaris* v2.1, retrieved from the Phytozome v.13 database [40], and quantified using STAR v.2 .7.10b [41] with the following parameters: --outFilterType BySJout --outFilterMultimapNmax 20 --alignSJoverhangMin 8 --alignSJDBoverhangMin 1 --outFilterMismatchNmax 999 --outFilterMismatchNoverLmax 0.10 --outFilterMatchNmin 50 --outFilterScoreMinOverLread 0 --outFilterMatchNminOverLread 0 --alignMatesGapMax 1000 --alignIntronMax 3000 --quantMode GeneCounts.

Differential expression analysis and enrichment of Gene Ontology terms

The differential expression analysis was conducted with the DESeq2 v.1.36.0 package [42] in R v.4.2.1 software [36], using the number of reads mapped to individual genes in the genome as estimated by STAR. The counts from the antisense strand were used to construct the counting matrix. Pre-filtering was performed to eliminate genes with very low expression levels (minimum overall read count of 10 reads). Multivariate analyses included hierarchical clustering using the average linkage method (UPGMA) based on the complement of Spearman correlations between the gene expression values of the samples, as well as principal component analysis (PCA).

The identification of DEGs was performed using raw count data for different contrasts, including comparisons between the two common bean cultivars at each of the three collection times and between different time points for each cultivar (Table 1). DEGs were determined when the p-value, adjusted for the false discovery rate (FDR) [43], was less than 0.05, and the log2 fold-change (based on the transcripts per million - TPM metric) was 2.0 or greater. Consequently, 6 contrasts were evaluated for the occurrence of DEGs (Table 1).

Table 1. Contrasts analyzed for differential gene expression considering the resistant (Ouro Vermelho) and susceptible (BRS Estilo) common bean cultivars to Lv134 of race 65 of *Colletotrichum lindemuthianum* in different collection times (0, 48, and 96 hai).

	Contrast	Up-regulated genes	Down-regulated genes
1	OV 48hai x E 48hai	Putative R48-genes[a]	Putative S48-genes[b]
2	OV 48hai x OV 0hai	Putative R48-genes[a]	Putative S48-genes[b]
3	E 48hai x E 0hai	Putative S48-genes[b]	Putative R48-genes[a]
4	OV 96hai x E 96hai	Putative R96-genes[c]	Putative S96-genes[d]
5	OV 96hai x OV 48hai	Putative R96-genes[c]	Putative S96-genes[d]
6	E 96hai x E 48hai	Putative S96-genes[d]	Putative R96-genes[c]

[a] R48-like genes: Intersection between Putative R48-genes; [b] S48-like genes: Intersection between Putative S48-genes

[c] R96-like genes: Intersection between Putative R96-genes; [d] S96-like genes: Intersection between Putative S96-genes

Genes regulated in contrasts 1 and 2 or downregulated in contrast 3 were considered putative resistance genes at 48 hai (putative R48-genes), while those downregulated in contrasts 1 and 2 or upregulated in contrast 3 were categorized as putative susceptible genes at 48 hai (putative S48-genes). The same reasoning was applied to contrasts 4, 5, and 6, as outlined in Table 1. Further analyses were conducted to identify overlaps between putative R-genes and S-genes, resulting in the identification of resistance-like genes (R48-like genes) and susceptibility-like genes (S48-like genes) at 48 hours after inoculation (hai). This methodology was also applied to derive R96-like genes and S96-like genes. The expression profiles of these genes, normalized to the log2 scale across the different treatments, were depicted using a heatmap organized according to a UPGMA clustering, performed using the R package pheatmap v.1.0.12 [44].

Gene functional profiling

Gene Ontology (GO) term enrichment analyses of the DEGs were performed using the topGO R v.2.48.0 package [45], applying Fisher's exact tests with an FDR adjusted p-value cut-off of 0.05. Based on gene annotations from Phytozome v.13 [46], the involvement of selected DEGs in potential metabolic pathways was explored. Enriched pathways among gene groups from the intersection of contrasts analyzed across different treatments were also investigated. For this purpose, the Enzyme Consortium (EC) numbers of enzymes associated with the genes of interest were used to identify the metabolic pathways in which these enzymes participate via the KEGG database [47]. Using this information, a Fisher's exact test (p-value of 0.05) was conducted in R statistical software to determine which pathways were enriched in groups of genes of interest.

Gene coexpression networks

To visualize the gene expression correlation patterns, coexpression networks were constructed using Pearson correlations (minimum correlation coefficient of 0.9) and the highest reciprocal rank (HRR) method [48]. A threshold of 30 correlations was set for selecting significant connections. Two coexpression networks were built using the R package igraph v.1.3.5 [49], one representing the susceptible genotype (BRS Estilo) and the other representing the resistant genotype (Ouro Vermelho). From the modeled networks, nodes related to the established DEG intersection groups were selected (S-like and R-like genes). To better understand the molecular interactions underlying resistance and susceptibility, the initial gene selection was extended to include the first-degree neighbors of these genes within the coexpression network. Additionally, hub scores for each gene were calculated using Kleinberg's hub centrality scores [50].

qPCR validation of differentially expressed genes

A total of 10 DEGs were randomly selected for validation using qPCR, in addition to the actin gene (Phvul.011G064500.1) as a normalizer (S1 Table). The sequences of these genes were obtained from Phytozome v13 (https://phytozome-next.jgi.doe.gov/). Primers and probes were designed following appropriate recommendations for qPCR assays, using the Primer3Plus online tool (https://www.primer3plus.com/). Some settings were adjusted, including product size ranges (80 - 120 bp), primer size (18 - 22 nt), GC content (40 - 60%), concentration of divalent cations (1.5 mM), and dNTPs (0.6 mM). Additional advanced settings were configured to avoid hairpin and dimer formation between the designed primers.

For cDNA synthesis, 1µg of RNA from each sample was used along with 0.5 µL of random hexamers (100 pmol) and 1 µL of 10 mM dNTP mix. The samples were incubated on a thermocycler at 65°C for 5 minutes and then placed on ice for 3 minutes. Each sample then received 4 µL of 5X RT Buffer and 1 µL of Maxima H Minus Enzyme Mix, followed by incubation on a thermocycler for 10 minutes at 25°C, 30 minutes at 50°C, and 5 minutes at 85°C to terminate the reaction.

qPCR reactions were carried out using PrimeTime Gene ExpressionMaster Mix (Integrated DNA Technologies, Coralville, IA) according to the manufacturer's guidelines on a QuantStudio 3 Real-Time PCR system. Two technical replicates were included for each of the three biological replicates. To visualize the mean quantification cycle (Cq) across the samples, we corrected the Cq values using the values obtained for the actin gene. Pearson linear correlations were performed between the RNA-Seq estimated gene expression and the deltaCq values using the R v.4.2.1 software [36].

Additionally, to compare differences across contrasts, we employed normalized log2 fold change differences and presented them in the bar plots created using R.

Results

Reaction of the common bean lines to isolate Lv134 of race 65 of *C. lindemuthianum*

The first symptoms of anthracnose appeared 96 hai on the stems and veins of the abaxial leaf surface BRS Estilo cultivar plants (average score of 8.78). In contrast, Ouro Vermelho cultivar plants remained symptom-free (average score 1.27) throughout the trial (Fig 1A-C). In susceptible plants, lesions progressively worsened, leading to plant death within seven days. These results are consistent with the expected compatible and incompatible interactions between the pathogen and the susceptible and resistant genotypes, respectively.

Fig 1. Phenotypic evaluation of the susceptible cultivar BRS Estilo (A) and resistant cultivar Ouro Vermelho (B), seven days after inoculation with isolate Lv 134, race 65 of *Colletotrichum lindemuthianum*. Student's t-test applied to the mean anthracnose severity scores of the BRS Estilo and Ouro Vermelho bean cultivars inoculated with *Colletotrichum lindemuthianum*. Asterisk (*) indicates significance at 0.1% probability by the t-test.

RNA-Seq results

After filtering with Trimmomatic, the number of reads retained for the 12 RNA-Seq libraries ranged from approximately 17 to 35 million, with an average of about 30 million (S2 Table). The reads mapped against the reference common bean genome (*Phaseolus*

vulgaris v2.1) showed an average of 90% uniquely mapped reads. PCA indicated that all replicates of samples from both BRS Estilo and Ouro Vermelho clustered together when collected at the same time point (Fig 2A). The first two components explained approximately 79% of the total variation among the 12 samples. The dendrogram also showed the clustering of replicates for each sample, as well as the clustering of cultivars under the same collection time point (Fig 2B). The expression profiles of ten genes differentially expressed by the cultivars Ouro Vermelho and Estilo, at 0, 48 and 96 hours after inoculation were evaluated by qPCR (S1 Fig) and corroborated the RNA-Seq results. These results attest to the satisfactory quality of the data for subsequent analyses.

Fig 2. Principal component analysis indicating the level of similarity among expression patterns of the common bean genes in response to the pathogen *Colletotrichum lindemuthianum*. (B) Dendrogram obtained by the UPGMA method (Unweighted Pair Group Method using Arithmetic averages) from the gene expression levels of the different common bean samples.

Differentially expressed genes over the time course of infection

By analyzing the contrasts between different treatments at various hai, we evaluated the expression of 22,344 genes throughout the infection period (S3 Table). In total, we conducted 6 contrasts, identifying DEGs that were either negatively or positively regulated at 48 or 96 hai for each genotype (Figs 3A and 3F). Initially, all DEGs were considered potential candidates for association with plant resistance or susceptibility to the pathogen. However, we refined this list by examining their expression across different contrasts and time points from the beginning of infection [19].

Fig 3. Differentially expressed genes in contrasts between cultivars and times of inoculation with the pathogen; and relevant intersections for inferences about association

with the pathosystem. (A) Number of DEGs upregulated and downregulated in contrasts 1, 2, and 3. (B, C) Venn diagrams indicating putative R48-genes or (D, E) putative S48-genes. (F) Number of downregulated and upregulated DEGs in contrasts 4, 5, and 6. (G, H) Venn diagrams indicating putative R96-genes or (I, J) putative S96-genes.

In contrast 1 between the genotypes Ouro Vermelho (R) and BRS Estilo (S) at 48 hai (OV 48 hai x E 48 hai), 770 DEGs were identified, with 353 being downregulated and 417 upregulated in the resistant cultivar. In contrast 2 (OV 48 hai x OV 0 hai), which considers the temporal change of gene expression in the resistant genotype, 6,474 DEGs were found, with 3,742 downregulated and 2,732 upregulated. In contrast 3 (E 48 hai x E 0 hai), reflecting the temporal change in the susceptible genotype, 2,761 were downregulated and 2,094 upregulated, totaling 4,855 DEGs (Fig 3A). Similar measurements were carried out in treatments at 96 hai (Fig 3F).

By performing specific intersections, we refined the association of genes with compatible or incompatible responses in this pathosystem. At 48 hai, for instance, 88 genes were positively regulated in both contrasts 1 and 2 (Fig. 3B), while 108 genes were upregulated in contrast 1 and downregulated in contrast 3 (Fig. 3C), thus identified as putative R48-genes (S4 Table). Conversely, 101 genes were negatively regulated in both contrasts 1 and 2 (Fig. 3D), while another 101 genes were downregulated in contrast 1 and upregulated in contrast 3 (Fig. 3E), regarded as putative S48-genes (S5 Table).

Similarly, at 96 hai, 620 genes were positively regulated in both contrasts 4 and 5 (Fig. 3G), while 105 genes were upregulated in contrast 4 and downregulated in contrast 6 (Fig. 3H), and were thus identified as putative R96-genes (S6 Table). On the other hand, 174 genes were negatively regulated in both contrasts 4 and 5 (Fig. 3I), while 193 genes were downregulated in contrast 4 and upregulated in contrast 6 (Fig. 3J), identified as

putative S96-genes (S7 Table). This approach further refines the repertoire of DEGs potentially associated with the pathosystem across different time points.

Samples were clustered using normalized expression values of genes identified as putative R genes and putative S genes (Fig 3). The heatmap provided a comprehensive view of gene expression profiles. Notably, genes previously unexpressed or downregulated at 48 hai in Ouro Vermelho were upregulated at 96 hai, indicating significant transcriptional changes during infection. The work by Padder et al., (2016) suggests a reprogramming of gene expression, which is like the results of our study. In the susceptible genotype BRS Estilo, putative S genes are upregulated, whereas putative R genes are downregulated at 48 hai. These results show how responses can progress to 96 hai, triggering a transition in gene expression regulation, with putative R genes switching from negative to positive regulation (Fig 4).

Fig 4. Hierarchical cluster analysis of normalized gene expression patterns in Ouro Vermelho and BRS Estilo. The heatmap shows the expression levels of significant differentially expressed genes within putative R-genes (A) and the putative S-genes (B) groups in plants inoculated with isolate Lv 134 of race 65 of *Colletotrichum lindemuthianum* at 0, 48 and 96 hai. The positive and negatively regulated genes are represented in red and blue, respectively.

Although we significantly reduced the number of DEGs associated with the pathosystem, we suggest additional filtering. We consider that genes associated with resistance should be up-regulated in contrasts 1 and 2. However, it would further corroborate if these genes were also downregulated in contrast 3, i.e., in the susceptible cultivar (S8 Table; Fig 5A-D). At 48 hai, three genes met these criteria and were considered resistance-like genes at 48 hai (R48-like genes). Likewise, eight genes were

found to be negatively regulated in contrasts 1 and 2, as well as positively regulated in contrast 3, and were therefore considered susceptibility-like genes (S48-like genes). For the 96 hai treatment, nine genes met analogous requirements to be considered R96-like genes, while seven were identified as S96-like genes.

Fig 5. Upset plots used to highlight shared genes between 3 contrasts and specific conditions to indicate R48-like genes (A), S48-like genes (B), R96-like genes (C), and S96-like genes (D).

Functional Profiling

GO enrichment analysis revealed distinct functional roles for the DEGs in the groups of putative R-genes and S-genes. DEGs considered as putative R48 genes and putative S48 genes were enriched for six and seven GO terms of biological processes, respectively (S9 Table). “Response to biotic stimulus” (GO:0009607) in both genotypes, as observed by [30], can indicate the presence of the pathogen. Following this, the significant representation of the “metabolic process” (GO:0008152) suggests the transcriptional reprogramming of plants in the manifestation of resistance. Additionally, “photosynthesis, light harvesting” (GO:0009765) and the “cellulose biosynthetic process” (GO:0030244) must be highlighted, as they reflect responses in terms of strengthening the resistant plant. Among the putative S48-genes, in addition to the “response to biotic stimulus” (GO:0009607), enrichments of the “defense response” (GO:0006952) and “response to oxidative stress” (GO:0006979) were observed, which may reflect the stress brought by the pathogen to the plant. Furthermore, the dynamics of the plant's response to stress include the “protein insertion into mitochondrial outer membrane” (GO:0045040), the “cellular glucan metabolic process” (GO:0006073), the “cell wall modification” (GO:0042545) and “protein ubiquitination” (GO:0016567). As

pathogen infection progressed to 96 hai, the "cellulose biosynthesis process" (GO: 0030244), present in the set of putative R48 genes, was the only process that remained among the putative R96 genes. Among the putative S96-genes, the "cell wall macromolecule catabolic process" (GO:0016998) stood out, while the "cell wall modification" (GO:0042545) was emphasized among the putative S48-genes. These results confirm distinct transcriptional dynamics between plants that are susceptible and those that are resistant to the pathogen.

We conducted an evaluation of enriched metabolic pathways and identified the KEGG pathways enriched in the putative R-genes group and the S-genes group (S10 Table). In the putative R-genes group, enriched pathways (p-value $\leq$ 0.01) included the phenylpropanoid biosynthesis pathway, represented by the arogenate dehydratase genes Phvul.006G149600 and Phvul.005G127100, which are positively regulated in the resistant genotype; the linoleic acid metabolism pathway, evidenced by the expression of lipoxygenase genes such as Phvul.006G185300, Phvul.005G156800, and Phvul.009G262900; and the glycerolipid metabolism pathway, indicated by the expression of glycerol-3-phosphate acyltransferase genes, for example, Phvul.002G192000. For the putative -genes group (p-value $\leq$ 0.01), in addition to the phenylpropanoid biosynthesis pathway, we also found enrichment in the biosynthesis pathways for flavonoids, tropane, piperidine and pyridine alkaloids, sesquiterpenoids and triterpenoids.

Gene coexpression networks

To provide further insights into the R-gene and S-gene sets, we modeled different gene coexpression networks to contrast the gene association patterns within each genotype. In total, four networks were constructed: (i) a network for Ouro Vermelho with the putative

R-genes (S11 Table); (ii) a network for BRS Estilo with the putative R-genes (S12 Table); (iii) a network for Ouro Vermelho with the putative S-genes (S13 Table); and (iv) a network for BRS Estilo with the putative S-genes (S14 Table).

In the resistant genotype, the coexpression network of putative R-genes showed the highest degree of interconnectivity among genes (S11 Table; Fig 6A). Hub genes within this network likely play an essential role during interaction with *C. lindemuthianum*. Notably, Phvul.002G106300 gene, encoding a GDSL-like Lipase/Acylhydrolase protein involved in signaling modulation pathways, had the highest number of connections (130 connections) and was more expressed in the resistant genotype compared to the susceptible genotype at 96 hai. Similarly, the Phvul.002G076400 gene, encoding a matrix metalloproteinase protein, ranked second in connectivity (116 connections) and exhibited differential upregulation at 96 hai in the resistant genotype. Phvul.002G107900 gene, encoding a pathogenesis-related protein like thaumatin, had 112 connections and was also more expressed at 96 hai in the resistant genotype than susceptible genotype. Genes associated with pathogen recognition, such as those encoding protein kinases (exemplified by the Phvul.002G115800, Phvul.002G115900, and Phvul.003G024000 genes) and NBS-LRR proteins (e.g. Phvul.002G021700) were also well-represented in the network with high connection values (106, 94, 52, and 50, respectively). Interestingly, these DEGs are concentrated on chromosomes two and three of the common bean genome. In fact, Jurado et al. (2022) reported that chromosomes Pv01, Pv02, Pv03, Pv04, Pv07, Pv08, and Pv11 have anthracnose-resistant loci. DEGs, such as Phvul.009G080000 (WRKY transcription factor) and Phvul.005G124100 (bZIP transcription factor), positively regulated at 48 hai in the resistant genotype, were present in the coexpression network of the R genes, as

well as genes associated with secondary carbohydrate metabolism, such as the enzyme beta glucosidase (Phvul.005G082800 - 96 hai).

Fig 6. Co-expression networks of putative R-genes and putative S-genes groups identified in different contrasts considering the times 0, 48 and 96 after inoculation of the Ouro Vermelho (resistant) and BRS Estilo (susceptible) cultivars with isolate Lv 134 of race 65 of *Colletotrichum lindemuthianum*. (A) putative R-genes obtained in contrasts from the resistant genotype; (B) putative R-genes obtained in contrasts from the susceptible genotype; (C) putative S-genes obtained in contrasts from the resistant genotype; (D) putative S-genes obtained in contrasts from the susceptible genotype.

Within the coexpression network of putative R-genes of the susceptible genotype (S12 Table), genes Phvul.002G106300 and Phvul.002G022600, encoding GDSL-like Lipase/Acylhydrolase proteins, also presented high connection values (82 and 59 degrees). Additionally, the Phvul.002G152700 gene, which encodes the enzyme leucoanthocyanidin dioxygenase related to flavonoid biosynthesis, had a highly connected network (78 degrees). It is necessary to consider these genes given the possibility that a pathway may be activated in different pathosystems. The isoflavone biosynthesis pathway is associated with bean resistance to common bacterial blight [52].

Considering the putative S-genes network in the resistant genotype, the central gene (102 connections) was Phvul.002G096500, which corresponds to a UDP-glucosyl transferase protein (S13 Table). In the putative S-genes network of the susceptible genotype, Phvul.002G073100 gene, encoding a MtN21 /EamA-like nodulin protein involved in sugar transport (71 degrees), was positively regulated in the susceptible genotype compared to the resistant one at 48 hai. Phvul.002G154600 gene, encoding a MATE efflux protein related to transmembrane transport of substances, was also more

expressed in the susceptible genotype at 48 hai and presented 66 connections in its network. The gene with the highest number of connections in this network was Phvul.002G096500, with 125 connections. It encodes a UDP-glucosyl transferase protein and was positively regulated in the susceptible genotype compared to the resistant genotype.

Differentially expressed genes located near previously identified loci associated with resistance to race 65 of *Colletotrichum lindemuthianum*

Previous studies have demonstrated the association of common bean loci *Co*-01, *Co*-02, *Co*-03, *Co*-04, *Co*-05, *Co*-06 and *Co*-15 with resistance to race 65 of *C. lindemuthianum* [5,14,15,17,18,53]. Genes located near these loci within the coexpression network in the resistant genotype that also showed differential expression patterns between the resistant and susceptible cultivars were investigated.

The Co-1 locus was previously mapped to the 49,583,965 base pair (bp) region of the Pv01 chromosome using the ss715645251 marker [9]. In our study, a total of 13 DEGs were identified within a 500 Kb window flanking this locus. Only one of these DEGs (Phvul.001G243300) was identified within the coexpression network of putative R-genes, while the other 12 genes were not part of any coexpression network. The Phvul.001G243300 gene, which encodes a C2 phospholipase, was exclusively expressed in the resistant genotype at 96 hai compared to 48 hai.

For the *Co*-04 locus, located on Pv08 and associated with the SAS13 marker (2,281,755 bp) [54], four DEGs encoding RLK-LRR proteins with malectin domains

were found. One of them, Phvul.008G028400, was upregulated at 96 hai in the resistant genotype and integrated into the coexpression network of putative R-genes.

Close to the *Co*-06 locus (marker SZ04) on chromosome Pv07 [55], one of the 54 DEGs identified was differentially expressed between the contrasts analyzed in this study. The Phvul.007G095300 gene, coding for the GATA transcription factor, was positively regulated only in the resistant genotype at 96 hai.

Discussion

In this study, we investigated compatible and incompatible interactions between common bean cultivars and *C. lindemuthianum* race 65 (isolate Lv134) at 0, 48 and 96 hai. Race 65 was chosen due its widespread distribution, high genetic and virulence diversity, and relevance to common bean breeding programs in Brazil [5,12,13,56,57]. The time points of 0, 48, and 96 hai were selected based on key steps in the infection process. The first visible symptoms in the susceptible cultivar (BRS Estilo) appeared approximately 96 hai (Fig 1), a pattern already observed by [28] with race 65 of the pathogen.

After inoculation, the fungus' conidia adhere to the surface of the plant, releasing germ tubes that develop into specialized structures called appressoria, which facilitate penetration into the plant's cuticle. This is followed by the formation of infection vesicles and differentiation of primary hyphae, characterizing the biotrophic phase of the fungus. During the biotrophic phase, which typically lasts approximately 24 hours, the host cell remains alive [58]. Subsequently, within 48 hours of post-penetration, the fungus transitions to the necrotrophic phase, characterized by the formation of secondary hyphae and initiation of host cell death. However, macroscopic symptoms only became visible

from 96 hai [58,59]. These time points enabled us to examine the dynamics of gene expression in plants in response to various stages of the pathogen's infection cycle.

To assess the alterations in gene expression patterns induced in this pathosystem, we analyzed the transcriptomes from the leaves of plants that are either resistant or susceptible to race 65 of *C. lindemuthianum*, utilizing six contrasts (Table 1). The analyses uncovered variations in gene expression patterns, offering a comprehensive understanding of the molecular mechanisms that contribute to plant resistance or susceptibility. Intersections between contrasts involving resistant and susceptible genotypes allowed the identification of DEGs that potentially contribute to the plant's response to the pathogen.

Initially, 88 genes upregulated in contrasts 1 and 2 (Fig 3B; S4 Table) were considered putative R48 genes, as were the 108 genes upregulated in contrast 1 and downregulated in contrast 3 (Fig 3C; S4 Table). On the other hand, 101 genes downregulated in contrasts 1 and 2 (Fig 3D), as well as another 101 genes downregulated in contrast 1 and upregulated in contrast 3 (Fig 3E), were considered putative S48 genes.

Among the putative R48 genes, notable examples include alpha-beta hydrolases, responsible for various catalytic reactions, disease resistance proteins containing the NB-ARC domain, and proteins with kinase domains, also identified in other *C. lindemuthianum-Phaseolus vulgaris* pathosystems. Among the putative S48-genes, we noted disease resistance proteins containing NB-ARC domains, TIR-NBS-LRR, RLK-LRRs, responsible for transducing signals among other functions [22], concanavalin A-type lectin family protein kinases, and integrase-type DNA-binding proteins.

Notable DEGs among the putative R96 genes (Fig 3F-J) include those encoding proteins from the superfamilies of protein kinases, bHLH, and sulfotransferase 2A.

Among the putative S96 genes, DEGs from the families of disease-resistant proteins containing the NB-ARC domain, RING/U-box proteins, and RLK-LRRs were identified. Several genes with such annotations have already been reported in other studies [29,60,61].

Our study identified a diversity of genes and functions in the sets of putative R and S-genes that suggest complex responses to the pathogen. To improve the identification of candidate genes, we investigated genes positively regulated in contrasts 1 and 2 and negatively regulated in contrast 3 (R48-like genes). It is plausible to assume that a gene that effectively contributes to the resistance of a resistant genotype to the pathogen should be inactive or downregulated in the susceptible genotype. Similarly, we looked for genes downregulated in contrasts 1 and 2 and upregulated in contrast 3 (S48-like genes). From this analysis, three genes were classified as R48-like genes, eight genes as S48-like genes, nine R96-like genes, and seven S96-like genes (Fig 5A-D).

Two of the R48-like genes were identified as hydrolases (Phvul.002G332300 and Phvul.L004500), and the third (Phvul.008G277466) belonged to the P450 family. Hydrolases perform catalytic roles in the hydrolysis of various bonds, participating in both primary and secondary metabolism, including lignin biosynthesis [60] and cell wall modification [62]. P450 family proteins are involved in the biosynthesis of lignin intermediates and other secondary metabolites, playing important roles in organism interactions, signal molecules, and defense against pathogens [63]. Among the S48-like genes, Phvul.005G001000 is associated with cellulose synthesis [64], while Phvul.004G129600 and Phvul.009G248400 are linked to protease inhibition [65] and secretion pathways [66], respectively, based on their PFAM description.

Two transcription factors from the PLATZ and WRKY families are notable among the R96-like genes, specifically Phvul.002G061300 and Phvul.005G181800, which have been highlighted in different transcriptomes [29,67]. Among the seven S96-like genes, Phvul.009G066300, Phvul.002G144600, and Phvul.001G145600 stand out. According to their annotations, they are directly or indirectly associated with lignin biosynthesis [68–70]. These findings suggest that both compatible and incompatible interactions can stimulate the plant at the cell wall level, warranting further investigation into metabolic pathways.

Recognizing that examining individual genes may not fully elucidate a genotype's resistance or susceptibility to *C. lindemuthianum*, we conducted functional analyses, including Gene Ontology (GO) annotations and metabolic pathway evaluations. The findings indicate that several differentially expressed genes (DEGs) within the sets of putative R-genes and putative S-genes may play significant roles in the mechanisms of resistance and susceptibility, respectively (S9 Table).

Plants recognize pathogens through extracellular receptors known as pattern recognition receptors (PRRs). Protein kinases, such as those encoded by the Phvul.003G024000, Phvul.002G115800, and Phvul.002G115900 genes, constitute the largest group of these receptors and are essential in the resistance process[18,22]. Protein kinases also mediate cell communication by autophosphorylation and downstream transphosphorylation of other proteins [71]. Thus, their expression is expected throughout the pathogen infection cycle. In our study, its expression was enhanced in the resistant genotype at 96 hai, indicating the relevance of the protein phosphorylation process (e.g. genes Phvul.003G024000, Phvul.002G115800, and Phvul.002G115900).

Upon successful delivery of its effector into the host cell, pathogen recognition occurs through specific interaction with NBS-LRR class proteins. In this study, these proteins were expressed in both genotypes at 48 hai, with a higher activation in the resistant genotype compared to the susceptible genotype (S3 Table). This class of proteins was well represented in the coexpression network of putative R-genes in the resistant genotype. The activation of these receptors in the common bean-*C. lindemuthianum* pathosystem has been previously reported by [30] and [29].

Pathogen recognition at the infection site triggers a systemic plant defense response via a signal transduction cascade, priming distal plant cells for an enhanced and coordinated defense [72]. Salicylic acid (SA), a key signaling molecule, plays a crucial role in mediating resistance responses. SA and phytoalexins, derived from the phenylpropanoid pathway [73], are essential for this process. Genes such as Phvul.006G149600 and Phvul.005G127100 encode arogenate dehydratase, a precursor enzyme in this pathway, and were part of the coexpression network of putative R-genes in the resistant genotype. In a similar way, the resistance of *Glycine max* to *Phytophthora sojae* has been linked to the activation of this pathway [74]. The importance of this pathway in the common bean-*C. lindemuthianum* pathosystem has been reported in previous studies for other races of fungus based on phenylalanine ammonia-lyase (PAL2) expression analyses [24–26,28].

The jasmonic acid (JA) pathway is another critical signaling pathway. JA is derived from α-linoleic acid, an important natural fatty acid. In our study, the linoleic acid production pathway was enriched in the putative R-genes set. Genes such as Phvul.006G185300, Phvul.005G156800, and Phvul.009G262900, which encode lipoxygenases, are involved in this pathway and were differentially expressed in the resistant genotype at 96 hai, with Phvul.009G262900 also differentially expressed at 48

hai. The association of lipoxygenase with plant responses to pathogens has been observed in different pathosystems. For example, increased lipoxygenase activity has been reported in resistant tobacco and rice plants infected with bacterial soft rot and *Magnaporthe oryzae*, respectively [75,76]. Another highly correlated gene in the putative R-genes network, Phvul.002G106300, belongs to the GDSL-like Lipase/Acylhydrolase family. Overexpression of this gene in *Arabidopsis thaliana* has been shown to improve resistance to the fungus *Sclerotinia sclerotiorum* by modulating pathways involving both SA and JA [77].

This study also identified the glycerolipid metabolism pathway as a potential component of the resistance mechanism of common bean plants to race 65 of *C. lindemuthianum*. Glycerol-3-phosphate and oleic acid are two important signaling molecules associated with resistance to fungal pathogens in plants. Li et al. (2020) observed that resistance of wheat to powdery mildew can be induced by glycerol through the regulation of plant fatty acid metabolism, promoting a cross-talk between phytohormones with JA and SA, and inducing the expression of pathogenesis-related genes.

Retrograde signaling is another communication pathway in plants. Through this mechanism, reactive oxygen species (ROS) produced in peroxisomes and other organelles activate signal transduction pathways, triggering rapid and adaptive responses. Additionally, peroxisomes synthesize phytohormones such as indole-3-acetic acid, JA, and SA [79]. Interestingly, the analysis of the putative R-genes set at 48 hai in the resistant genotype revealed enrichment of biological processes related to peroxisome fission (S9 Table). Furthermore, the second most connected gene in the coexpression network of putative R-genes in the resistant genotype encodes a matrix metalloproteinase. While its precise role in plant defense remains under investigation, similar genes in tomato function

as positive regulators of defense against *Botrytis cinerea* by controlling ROS accumulation [80].

Transcription factors have been associated with the defense response to biotic stimuli [81–83]. Positive regulation of WRKY and bZIP TFs was observed in the resistant genotype at 48 hai, and they were also present in the coexpression network of putative R-genes. It should be noted that transcription factors WRKY (Phvul.005G181800) and PLATZ (Phvul.002G061300) were classified as R96-like genes, which means that they were positively regulated in contrasts 4 and 5 and negatively regulated in contrast 6. The WRKY TFs are associated with SA and ethylene signaling pathways, phytohormones that were highlighted in the study by [30] with the pathosystem *C. lindemuthianum-P. vulgaris*, as well as in the response to infection of *Botrytis cinerea* and *Aspergillus flavus* in *Arabidopsis thaliana* and *Zea mays*, respectively [81,82]. Padder et al. (2016) have also identified high expression of different transcription factors in common beans after infection with race 73 of *C. lindemuthianum*, observing up-regulation of WRKY in the susceptible genotype as well.

Cell signaling mechanisms induce the synthesis of pathogenesis-related (PR) proteins, which are well-characterized components of plant defense responses, often exhibiting antimicrobial or enzymatic activities that directly target pathogens [84]. In our study, three major latex-like (MLP) genes (Phvul.011G183500, Phvul.011G183766, and Phvul.011G183832), members of the PR family, were identified within the network of putative R-genes of the resistant genotype. They were also represented by the annotation of the biological process “response to biotic stimuli” in the putative R-genes at 48 and 96 hai and in the putative S-genes at 48 hai (S9 Table). These proteins correspond to a subfamily of the Bet v_1 protein family. Members of this family respond to biotic and abiotic stresses and play important roles in plant growth and development, including

disease resistance and stress tolerance [85]. According to [86], MLP 28 protein enhances resistance to *Potato virus* Y in *Nicotiana benthamiana*. Another PR gene that showed significant association with other genes in the coexpression network of putative R-genes was the gene encoding thaumatin-like pathogenesis protein (Phvul.002G107900), which has also been associated with wheat resistance to the fungus *Puccinia triticina* [87].

Upon pathogen infiltration of the cell wall, plants synthesize new carbohydrates to reinforce the compromised wall [88]. In this study, genes encoding cellulose such as Phvul.002G136300 (at 96 hai) and Phvul.005G001000 (at 48 and 96 hai) were up-regulated in the resistant genotype. Genes with glycosyltransferase activity are essential in the context of cell wall synthesis [64]. The process associated with cellulose metabolism was enriched in the putative R48-genes and putative S48-genes. However, it was maintained over time only in the putative R-genes set in the resistant genotype, suggesting a more robust and prolonged response to the pathogen. Susceptible hosts typically exhibit deficiencies in the papilla development and secondary wall thickness, potentially weakening their ability to resist pathogen invasion [89]. Beyond cellulose, the resistant genotype exhibited upregulated expression of genes associated with xyloglucan endotransglucosylase/hydrolase (Phvul.003G147300 and Phvul.003G231900), suggesting increased glucan deposition in the cell wall. Additionally, genes involved in pectin modification (Phvul.005G011900, Phvul.005G184400, Phvul.009G222100, and Phvul.010G123132) were upregulated in the resistant genotype, potentially contributing to enhanced cell wall structure and function. On the other hand, resistant plants showed downregulation of genes associated with carbohydrate metabolism, such as beta-glucosidase enzymes and hydrolases (Phvul.005G082800, Phvul.005G130900), compared to susceptible plants. This reduced activity in processes associated with

primary metabolism and photosynthesis likely frees up energy resources that the plant can reallocate towards its defense response [90].

Focusing on the putative R-genes in the susceptible genotype, genes encoding SAUR-type auxin responsive protein, such as Phvul.006G022900 and Phvul.009G001400, were present in its coexpression network. Enhanced auxin signaling is known to promote disease development in certain plant-pathogen interactions. The presence of free auxin increases in plants infected with the fungal *Puccinia graminis*, as well as with bacteria *Pseudomona syringae*, *Xanthomonas campestris,* and *Ralstonia solanacearum* [91–96]. Additionally, sugar transporters encoded by Phvul.002G073100, Phvul.001G129500, and Phvul.007G083300 were also up-regulated at 96 hai in the susceptible genotype and are part of the network of putative S-genes of the susceptible genotype. Padder et al. (2016) also verified the expression of the Phvul.009G001400 (SAUR-like auxin-responsive) and Phvul.001G129500 genes, aligning with our findings. These proteins may contribute to the survival of the fungus in the plant, as several sugar-breaking enzymes are crucial for the maintenance of the fungus in necrotrophic lesions [29]. The expression of nodulin-like genes, known to transport sucrose, is important for pathogens to improve their fitness during host colonization [92].

In the set of putative S-genes, in addition to the activation of the phenylpropanoid pathway, two significantly enriched pathways were associated with the biosynthesis of secondary metabolites (including flavonoid, isoflavonoid, tropane alkaloid, piperidine, and pyridine) and biosynthesis of sesquiterpenoid and triterpenoid. These pathways enable the production of phytoalexins, which are defined as low-weight antimicrobial metabolites synthesized by plants in response to a pathogen infection [97]. However, because the colonization of plants by pathogenic microorganisms is a spatially dynamic process, the effective contribution of secondary metabolites to plant defense depends on

the accumulation of sufficient quantities of these products in the right places and at the right times [98]. The central gene of the network of putative S-genes (102 connections) was Phvul.002G096500, which encodes a UDP-glucosyl transferase protein. The expression of glucosyltransferase is induced by deoxynivalenol, as well as salicylic acid, ethylene and jasmonic acid [99]. The biological process associated with glutamate biosynthesis was noted among the putative S-genes (S9 Table). During interactions with pathogens, glutamate metabolism can lead to two metabolic states: "resistance", in which cell viability is maintained, and "evasion", in which the process of cell death is facilitated. Hemibiotrophic pathogens exploit this metabolic vulnerability to manipulate glutamate metabolism, promoting both their establishment and persistence within the host [100].

Different loci have been associated with the response of common bean to race 65 of *C. lindemuthianum* [14–18,53]. In this study, DEGs located close to *Co*-1, *Co*-4, and *Co*-6 loci, present on the Pv01, Pv08, and Pv11 chromosomes of *P. vulgaris*, respectively, were verified. Close to *Co-1* locus, a gene encoding a phospholipase C was upregulated in the resistant genotype and was part of its coexpression network of putative R-genes. This enzyme hydrolyzes the phosphodiester bond on the glycerol side of phospholipids to produce diacylglycerol (DAG) and a phosphorylated head group. DAG is subsequently phosphorylated by a diacylglycerol kinase into phosphatidic acid, a second messenger within cell signaling pathways triggered upon recognition of pathogen effectors [101]. The participation of phospholipid signaling in Arabidopsis in response to infection by *Pseudomonas syringae* is well documented [102]. A cluster of genes encoding kinases with malectin domains were located on the Pv08 chromosome; one of these genes was upregulated in the resistant genotype at 96 hai and was part of its coexpression network of putative R-genes. Such proteins function as cell wall sensors, and the detection of wall disturbances and consequent signaling are crucial for plant growth and survival [103].

[22] associated some proteins of this class in the same locations where anthracnose resistance loci have been identified in the common bean genome. Associated with the *Co*-06 locus, a DEG encoding a GATA transcription factor was up-regulated and present in the coexpression network of putative R-genes of the resistant genotype. The interaction between the transcription factor GATA and the jasmonic acid signaling pathway has been reported in the pathosystem *Brachypodium distachyon-Magnaporthe oryzae* [83].

The inheritance of resistance of Ouro Vermelho to Lv134, race 65, of *C. lindemuthianum* has been reported as monogenic [13]. Linkage mapping revealed SNP markers located on the Pv04 significantly associated with the resistance of Ouro Vermelho to this isolate of race 65. However, in the present study, genes located close to this region were not significantly differentially expressed between the two genotypes at 48 or 96 hai, nor were part of the coexpression networks. Further studies should evaluate differential gene expression at earlier and later time post-inoculation. Significant expression of genes located on the Pv02 chromosome, such as Phvul.002G332300 (alpha/beta-Hydrolase), Phvul.002G061300 (PLATZ transcription factor), and Phvul.002G142600 (hydroxyproline-rich glycoprotein), was identified in the present study, with many of them integrated into the coexpression networks of the resistant genotype. Two resistance loci have been identified on this chromosome (*Co*-u and *CoPv02c*^{X}) in the cultivars BAT 93 and Xana, respectively, associated with resistance to race 65 *of C. lindemuthianum* [9,10,53].

Our study investigated the dynamic transcriptional profiles of common bean genotypes during compatible and incompatible interactions with the isolate Lv 134, race 65, of *C. lindemuthianum.* Time-course RNA-Seq analysis at 0, 48, and 96 hai revealed significant transcriptional reprogramming associated with activation of different genes from different metabolic pathways. Potential candidate genes involved in common bean

resistance against this pathogen were identified, including their expression profiles across different infection stages and their interaction within coexpression networks. Considering the specificity of the genetic control of resistance in pathosystems involving different races, this study provides a valuable overview of the response of resistant and susceptible genotypes to race 65 of the pathogen, which is relevant in the economic scenario of common bean crop production.

Acknowledgements

We are grateful to the Brazilian funding agencies: Coordenadoria de Aperfeiçoamento de Pessoal de Nível Superior (CAPES) and Conselho Nacional de Desenvolvimento Científico e Tecnológico (CNPq). The authors would like to also thank Dr. Oscar P. Hurtado-Gonzáles from the US Department of Agriculture, Animal and Plant Health Inspection Service, for his essential support to the development of this study.

References

1. De Ron AM, González AM, Paula Rodiño A, Santalla M, Godoy L, Papa R. History of the common bean crop: its evolution beyond its areas of origin and domestication. Arbor. 2016;192: a317. doi:10.3989/arbor.2016.779n3007

2. Padder BA, Sharma PN, Awale HE, Kelly JD. *Colletotrichum lindemuthianum*, the causal agent of bean anthracnose. Journal of Plant Pathology. Edizioni ETS; 2017. pp. 317–330. doi:10.4454/jpp.v99i2.3867

3. Ferreira JJ, Campa A, Kelly JD. Organization of Genes Conferring Resistance to Anthracnose in Common Bean. Translational Genomics for Crop Breeding. Wiley; 2013. pp. 151–181. doi:10.1002/9781118728475.ch9

4. Flor HH. Current Status of the Gene-For-Gene Concept. Annu Rev Phytopathol. 1971;9: 275–296. Available: https://api.semanticscholar.org/CorpusID:85578295

5. Vidigal Filho PS, Gonçalves-Vidigal MC, Vaz Bisneta M, Souza VB, Gilio TAS, Calvi AA, et al. Genome-wide association study of resistance to anthracnose and angular leaf spot in Brazilian Mesoamerican and Andean common bean cultivars. Crop Sci. 2020;60: 2931–2950. doi:10.1002/csc2.20308

6. González VM, Yuste-Lisbona FJ, Rodiño AP, De Ron AM, Capel C, García-Alcázar M, et al. Uncovering the genetic architecture of *Colletotrichum lindemuthianum* resistance through QTL mapping and epistatic interaction analysis in common bean. Front Plant Sci. 2015;6. doi:10.3389/fpls.2015.00141

7. Zuiderveen GH, Padder BA, Kamfwa K, Song Q, Kelly JD. Genome-Wide Association Study of Anthracnose Resistance in Andean Beans (*Phaseolus vulgaris*). PLoS One. 2016;11: e0156391. doi:10.1371/journal.pone.0156391

8. Choudhary N, Bawa V, Paliwal R, Singh B, Bhat MohdA, Mir JI, et al. Gene/QTL discovery for Anthracnose in common bean (*Phaseolus vulgaris* L.) from North-western Himalayas. PLoS One. 2018;13: e0191700. doi:10.1371/journal.pone.0191700

9. Campa A, Rodríguez-Suárez C, Giraldez R, Ferreira JJ. Genetic analysis of the response to eleven *Colletotrichum lindemuthianum* races in a RIL population of common bean (*Phaseolus vulgaris* L.). BMC Plant Biol. 2014;14: 115. doi:10.1186/1471-2229-14-115

10. Geffroy V, Sévignac M, Billant P, Dron M, Langin T. Resistance to *Colletotrichum lindemuthianum* in *Phaseolus vulgaris*: a case study for mapping two independent genes. Theoretical and Applied Genetics. 2008;116: 407–415. doi:10.1007/s00122-007-0678-y

11. Gonçalves-Vidigal MC, Thomazella C, Vidigal Filho PS, Kvitschal MV, Elias HT. Characterization of *Colletotrichum lindemuthianum* isolates using differential cultivars of common bean in Santa Catarina State, Brazil. Brazilian Archives of Biology and Technology. 2008;51: 883–888. doi:10.1590/S1516-89132008000500002

12. Davide LMC, Souza EA. Pathogenic variability within race 65 of *Colletotrichum lindemuthianum* and its implications for common bean breeding. Cropp Breeding and Applied Biotechnology. 2009;9: 23–30. doi:10.12702/1984-7033.v09n01a04

13. Costa LC, Nalin RS, Ramalho MAP, de Souza EA. Are duplicated genes responsible for anthracnose resistance in common bean? PLoS One. 2017;12: e0173789. doi:10.1371/journal.pone.0173789

14. Costa LC, Nalin RS, Dias MA, Ferreira ME, Song Q, Pastor-Corrales MA, et al. Different loci control resistance to different isolates of the same race of *Colletotrichum lindemuthianum* in common bean. Theoretical and Applied Genetics. 2021;134: 543–556. doi:10.1007/s00122-020-03713-x

15. Lilia A-M, Sartorato A. Analyses of the pathogenic variability of *Colletotrichum lindemuthianum* in Brazil. Annual report of the Bean Improvement Cooperative. 2004;47: 241–242.

16. Vidigal Filho PS, Gonçalves-Vidigal MC, Kelly JD, Kirk WW. Sources of Resistance to Anthracnose in Traditional Common Bean Cultivars from Paraná, Brazil. Journal of Phytopathology. 2007;155: 108–113. doi:10.1111/j.1439-0434.2007.01203.x

17. Vieira AF, Almeida LCS, Rodrigues LA, Costa JGC, Melo LC, Pereira HS, et al. Research Article Selection of resistance sources to common bean anthracnose by field phenotyping and DNA marker-assisted screening. Genetics and Molecular Research. 2018;17. doi:10.4238/gmr18066

18. Vaz Bisneta M, Gonçalves-Vidigal MC. Integration of anthracnose resistance loci and RLK and NBS-LRR-encoding genes in the *Phaseolus vulgaris* L. genome. Crop Sci. 2020;60: 2901–2918. doi:10.1002/csc2.20288

19. Hoseinzadeh AH, Soorni A, Shoorooei M, Torkzadeh Mahani M, Maali Amiri R, Allahyari H, et al. Comparative transcriptome provides molecular insight into defense-associated mechanisms against spider mite in resistant and susceptible common bean cultivars. PLoS One. 2020;15: e0228680. doi:10.1371/journal.pone.0228680

20. Andersen EJ, Ali S, Byamukama E, Yen Y, Nepal MP. Disease Resistance Mechanisms in Plants. Genes (Basel). 2018;9: 339. doi:10.3390/genes9070339

21. Aono AH, Pimenta RJG, Dambroz CM da S, Costa FCL, Kuroshu RM, de Souza AP, et al. Genome-wide characterization of the common bean kinome: Catalog and insights into expression patterns and genetic organization. Gene. 2023;855: 147127. doi:10.1016/j.gene.2022.147127

22. da Silva Dambroz CM, Aono AH, de Andrade Silva EM, Pereira WA. Genome-wide analysis and characterization of the LRR-RLK gene family provides insights into anthracnose resistance in common bean. Sci Rep. 2023;13: 13455. doi:10.1038/s41598-023-40054-3

23. Boller T, Felix G. A Renaissance of Elicitors: Perception of Microbe-Associated Molecular Patterns and Danger Signals by Pattern-Recognition Receptors. Annu Rev Plant Biol. 2009;60: 379–406. doi:10.1146/annurev.arplant.57.032905.105346

24. Fraire-Velázquez S, Lozoya-Gloria E. Differential early gene expression in *Phaseolus vulgaris* to Mexican isolates of *Colletotrichum lindemuthianum* in incompatible and compatible interactions. Physiol Mol Plant Pathol. 2003;63: 79–89. doi:10.1016/j.pmpp.2003.10.004

25. Oblessuc PR, Borges A, Chowdhury B, Caldas DGG, Tsai SM, Camargo LEA, et al. Dissecting *Phaseolus vulgaris* Innate Immune System against *Colletotrichum lindemuthianum* Infection. PLoS One. 2012;7: e43161. doi:10.1371/journal.pone.0043161

26. Borges A, Melotto M, Tsai SM, Caldas DGG. Changes in spatial and temporal gene expression during incompatible interaction between common bean and anthracnose pathogen. J Plant Physiol. 2012;169: 1216–1220. doi:10.1016/j.jplph.2012.04.003

27. Fontenelle MR, Santana MF, Cnossen A, Bazzolli DMS, Bromonschenkel SH, de Araújo EF, et al. Differential expression of genes during the interaction between *Colletotrichum lindemuthianum* and *Phaseolus vulgaris*. Eur J Plant Pathol. 2017;147: 653–670. doi:10.1007/s10658-016-1033-4

28. da Silva CM, Costa LC, Porto ACM, Lima AA, Chalfun-Junior A, de Souza EA, et al. Differential gene expression in common bean during interaction with race 65 of *Colletotrichum lindemuthianum*. Trop Plant Pathol. 2021;46: 518–527. doi:10.1007/s40858-021-00447-z

29. Padder BA, Kamfwa K, Awale HE, Kelly JD. Transcriptome Profiling of the *Phaseolus vulgaris - Colletotrichum lindemuthianum* Pathosystem. PLoS One. 2016;11: e0165823. doi:10.1371/journal.pone.0165823

30. Alvarez-Diaz JC, Laugé R, Delannoy E, Huguet S, Paysant-Le Roux C, Gratias A, et al. Genome-Wide Transcriptomic Analysis of the Effects of Infection with the Hemibiotrophic Fungus *Colletotrichum lindemuthianum* on Common Bean. Plants. 2022;11: 1995. doi:10.3390/plants11151995

31. Tu JC. An improved Mathur's medium for growth sporulation, and germination of spores of *Colletotrichum lindemuthianum*. Microbios. 1985;44: 87–93.

32. Mathur RS, Barnett HL, Lilly VG. Sporulation of *Colletotrichum lindemuthianum* in culture. Phytopathology. 1950;40. Available: https://api.semanticscholar.org/CorpusID:81445520

33. Rava CA, Molina J, Kauffmann M, Briones I. Determinación de razas fisiológicas de *Colletotrichum lindemuthianum*un en Nicarágua. Fitopatol Bras. 1993; 388–391.

34. Burt AJ, William HM, Perry G, Khanal R, Pauls KP, Kelly JD, et al. Candidate Gene Identification with SNP Marker-Based Fine Mapping of Anthracnose Resistance Gene Co-4 in Common Bean. PLoS One. 2015;10: e0139450. doi:10.1371/journal.pone.0139450

35. van Schoonhoven A, Pastor-Corrales MA. Standard system for the evaluation of bean germoplasm. Cali, Colômbia: CIAT (Centro Internacional de Agricultura Tropical); 1987.

36. CoreTeam. R: A language and environment for statistical computing. In: Vol. 2 [Internet]. 2018 [cited 22 Mar 2023]. Available: https://www.r-project.org/

37. Miranda RN de, Silva CM da, Porto AC da M, Pereira WA. Protocol adjustment improves the extraction of high-quality total RNA from common bean stems infected by Sclerotinia sclerotiorum. Ciência e Agrotecnologia. 2019;43. doi:10.1590/1413-7054201943024618

38. Andrews S. FastQC: a quality control tool for high throughput sequence data. 2010 [cited 22 Mar 2023]. Available: https://www.bioinformatics.babraham.ac.uk/projects/fastqc/

39. Bolger AM, Lohse M, Usadel B. Trimmomatic: a flexible trimmer for Illumina sequence data. Bioinformatics. 2014;30: 2114–2120. doi:10.1093/bioinformatics/btu170

40. Schmutz J, McClean PE, Mamidi S, Wu GA, Cannon SB, Grimwood J, et al. A reference genome for common bean and genome-wide analysis of dual domestications. Nat Genet. 2014;46: 707–713. doi:10.1038/ng.3008

41. Dobin A, Davis CA, Schlesinger F, Drenkow J, Zaleski C, Jha S, et al. STAR: ultrafast universal RNA-seq aligner. Bioinformatics. 2013;29: 15–21. doi:10.1093/bioinformatics/bts635

42. Love MI, Huber W, Anders S. Moderated estimation of fold change and dispersion for RNA-seq data with DESeq2. Genome Biol. 2014;15: 550. doi:10.1186/s13059-014-0550-8

43. Benjamini Y, Hochberg Y. Controlling the False Discovery Rate: A Practical and Powerful Approach to Multiple Testing. J R Stat Soc Series B Stat Methodol. 1995;57: 289–300. doi:10.1111/j.2517-6161.1995.tb02031.x

44. Kolde R. Pheatmap: pretty heatmaps. R package version 1.2. 2012 [cited 22 Mar 2023]. Available: https://rdrr.io/cran/pheatmap/

45. Alexa A, Rahnenfuhrer J. topGO: Enrichment Analysis for Gene Ontology. R package version 2.50.0. 2022 [cited 13 Mar 2023]. Available: https://bioconductor.statistik.tu-dortmund.de/packages/3.3/bioc/vignettes/topGO/inst/doc/topGO.pdf

46. Goodstein DM, Shu S, Howson R, Neupane R, Hayes RD, Fazo J, et al. Phytozome: a comparative platform for green plant genomics. Nucleic Acids Res. 2012;40: D1178–D1186. doi:10.1093/nar/gkr944

47. Kanehisa M. KEGG: Kyoto Encyclopedia of Genes and Genomes. Nucleic Acids Res. 2000;28: 27–30. doi:10.1093/nar/28.1.27

48. Mutwil M, Usadel B, Schuئtte M, Loraine A, Ebenhoئh O, Persson S. Assembly of an Interactive Correlation Network for the Arabidopsis Genome Using a Novel Heuristic Clustering Algorithm . Plant Physiol. 2009;152: 29–43. doi:10.1104/pp.109.145318

49. Csárdi G, Nepusz T. The igraph software package for complex network research. 2006. Available: https://api.semanticscholar.org/CorpusID:16923281

50. Kleinberg JM. Hubs, authorities, and communities. ACM Comput Surv. 1999;31: 5. doi:10.1145/345966.345982

51. Jurado M, Campa A, Ferreira JJ. Differentially expressed genes against Colletotrichum lindemuthiamum in a bean genotype carrying the Co-2 gene revealed by RNA-sequencing analysis. Front Plant Sci. 2022;13. doi:10.3389/fpls.2022.981517

52. Cox LD, Munholland S, Mats L, Zhu H, Crosby WL, Lukens L, et al. The Induction of the Isoflavone Biosynthesis Pathway Is Associated with Resistance to Common Bacterial Blight in *Phaseolus vulgaris* L. Metabolites. 2021;11: 433. doi:10.3390/metabo11070433

53. Paulino PPS, Gonçalves-Vidigal MC, Bisneta MV, Vidigal Filho PS, Nunes MPBA, Xavier LFS, et al. Occurrence of anthracnose pathogen races and resistance genes in common bean across 30 years in Brazil. Agronomy Science and Biotechnology. 2021;8: 1–21. doi:10.33158/ASB.r140.v8.2022

54. Oblessuc PR, Francisco C, Melotto M. The Co-4 locus on chromosome Pv08 contains a unique cluster of 18 COK-4 genes and is regulated by immune response in common bean. Theoretical and Applied Genetics. 2015;128: 1193–1208. doi:10.1007/s00122-015-2500-6

55. Campa A, Trabanco N, Ferreira JJ. Identification of Clusters that Condition Resistance to Anthracnose in the Common Bean Differential Cultivars AB136 and MDRK. Phytopathology. 2017;107: 1515–1521. doi:10.1094/PHYTO-01-17-0012-R

56. Coelho M, Gonçalves-Vidigal MC, Sousa LL de, Nunes MPBA, Azevedo RF, Galván MZ. <b>Characterization of race 65 of <i>*Colletotrichum lindemuthianum*</i> by sequencing ITS regions. Acta Sci Agron. 2016;38: 429. doi:10.4025/actasciagron.v38i4.30586

57. Ribeiro T, Esteves JA de F, Silva DA, Gonçalves JGR, Carbonell SAM, Chiorato AF. Classificação de raças de *Colletotrichum lindemuthianum* em cultivares diferenciadoras de feijoeiro comum. Acta Sci Agron. 2016;38: 179. doi:10.4025/actasciagron.v38i2.27866

58. O'Connell RJ, Bailey JA, Richmond DV. Cytology and physiology of infection of *Phaseolus vulgaris* by *Colletotrichum lindemuthianum*. Physiol Plant Pathol. 1985;27: 75–98. doi:10.1016/0048-4059(85)90058-X

59. Skipp RA, Deverall BJ. Relationships between fungal growth and host changes visible by light microscopy during infection of bean hypocotyls (*Phaseolus vulgaris*) susceptible and resistant to physiological races of *Colletotrichum lindemuthianum*. Physiol Plant Pathol. 1972;2: 357–374. doi:10.1016/S0048-4059(72)80007-9

60. Mindrebo JT, Nartey CM, Seto Y, Burkart MD, Noel JP. Unveiling the functional diversity of the alpha/beta hydrolase superfamily in the plant kingdom. Curr Opin Struct Biol. 2016;41: 233–246. doi:10.1016/j.sbi.2016.08.005

61. Kuwabo K, Hamabwe SM, Kachapulula P, Cichy K, Parker T, Mukuma C, et al. Genome-wide association analysis of anthracnose resistance in the Yellow Bean Collection of Common Bean. PLoS One. 2023;18: e0293291. doi:10.1371/journal.pone.0293291

62. Mejía-Mendoza MA, Garcidueñas-Piña C, Padilla-Ramírez JS, Soria-Guerra RE, Morales-Domínguez JF. Identification in silico and expression analysis of a β-1-4-

endoglucanase and β-galactosidase genes related to ripening in guava fruit. Journal of Genetic Engineering and Biotechnology. 2022;20: 3. doi:10.1186/s43141-021-00289-x

63. Sun K, Fang H, Chen Y, Zhuang Z, Chen Q, Shan T, et al. Genome-Wide Analysis of the Cytochrome P450 Gene Family Involved in Salt Tolerance in Gossypium hirsutum. Front Plant Sci. 2021;12. doi:10.3389/fpls.2021.685054

64. Liu X, Zhang H, Zhang W, Xu W, Li S, Chen X, et al. Genome-wide bioinformatics analysis of Cellulose Synthase gene family in common bean (*Phaseolus vulgaris* L.) and the expression in the pod development. BMC Genom Data. 2022;23: 9. doi:10.1186/s12863-022-01026-0

65. Alves M, Chaves I, Carrilho D, Veloso M, Ricardo CP. Detection of novel trypsin inhibitors in the cotyledons of *Phaseolus vulgaris* seeds. J Plant Physiol. 2010;167: 848–854. doi:10.1016/j.jplph.2010.01.007

66. Khare D, Choi H, Huh SU, Bassin B, Kim J, Martinoia E, et al. *Arabidopsis* ABCG34 contributes to defense against necrotrophic pathogens by mediating the secretion of camalexin. Proceedings of the National Academy of Sciences. 2017;114. doi:10.1073/pnas.1702259114

67. Kamfwa K, Zhao D, Kelly JD, Cichy KA. Transcriptome analysis of two recombinant inbred lines of common bean contrasting for symbiotic nitrogen fixation. PLoS One. 2017;12: e0172141. doi:10.1371/journal.pone.0172141

68. Liu C, Qiao X, Li Q, Zeng W, Wei S, Wang X, et al. Genome-wide comparative analysis of the BAHD superfamily in seven Rosaceae species and expression analysis in pear (Pyrus bretschneideri). BMC Plant Biol. 2020;20: 14. doi:10.1186/s12870-019-2230-z

69. Barakat A, Bagniewska-Zadworna A, Choi A, Plakkat U, DiLoreto DS, Yellanki P, et al. The cinnamyl alcohol dehydrogenase gene family in Populus: phylogeny, organization, and expression. BMC Plant Biol. 2009;9: 26. doi:10.1186/1471-2229-9-26

70. Cho JY, Choi GJ, Son SW, Jang KS, Lim HK, Lee SO, et al. Isolation and antifungal activity of lignans from *Myristica fragrans* against various plant pathogenic fungi. Pest Manag Sci. 2007;63: 935–940. doi:10.1002/ps.1420

71. Hubbard SR, Miller WT. Receptor tyrosine kinases: mechanisms of activation and signaling. Curr Opin Cell Biol. 2007;19: 117–123. doi:10.1016/j.ceb.2007.02.010

72. Dangl JL, Jones JDG. Plant pathogens and integrated defence responses to infection. Nature. 2001;411: 826–833. doi:10.1038/35081161

73. Yadav V, Wang Z, Wei C, Amo A, Ahmed B, Yang X, et al. Phenylpropanoid Pathway Engineering: An Emerging Approach towards Plant Defense. Pathogens. 2020;9: 312. doi:10.3390/pathogens9040312

74. Li P, Liu D, Hu J, Yu B. <scp>RNA-seq</scp> analysis of soybean (*Glycine max*) responding to *Phytophthora sojae*. Journal of Phytopathology. 2023;171: 180–188. doi:10.1111/jph.13166

75. Yara A, Yaeno T, Montillet J-L, Hasegawa M, Seo S, Kusumi K, et al. Enhancement of disease resistance to Magnaporthe grisea in rice by accumulation of hydroxy linoleic acid. Biochem Biophys Res Commun. 2008;370: 344–347. doi:10.1016/j.bbrc.2008.03.083

76. Sumayo MS, Kwon D-K, Ghim S-Y. Linoleic acid-induced expression of defense genes and enzymes in tobacco. J Plant Physiol. 2014;171: 1757–1762. doi:10.1016/j.jplph.2014.08.015

77. Ding L, Li M, Guo X, Tang M, Cao J, Wang Z, et al. Arabidopsis GDSL1 overexpression enhances rapeseed Sclerotinia sclerotiorum resistance and the functional identification of its homolog in Brassica napus. Plant Biotechnol J. 2020;18: 1255–1270. doi:10.1111/pbi.13289

78. Li Y, Qiu L, Liu X, Zhang Q, Zhuansun X, Fahima T, et al. Glycerol-Induced Powdery Mildew Resistance in Wheat by Regulating Plant Fatty Acid Metabolism, Plant Hormones Cross-Talk, and Pathogenesis-Related Genes. Int J Mol Sci. 2020;21: 673. doi:10.3390/ijms21020673

79. Su T, Li W, Wang P, Ma C. Dynamics of Peroxisome Homeostasis and Its Role in Stress Response and Signaling in Plants. Front Plant Sci. 2019;10. doi:10.3389/fpls.2019.00705

80. Li D, Zhang H, Song Q, Wang L, Liu S, Hong Y, et al. Tomato Sl3-MMP, a member of the Matrix metalloproteinase family, is required for disease resistance against Botrytis cinerea and Pseudomonas syringae pv. tomato DC3000. BMC Plant Biol. 2015;15: 143. doi:10.1186/s12870-015-0536-z

81. Birkenbihl RP, Diezel C, Somssich IE. Arabidopsis WRKY33 Is a Key Transcriptional Regulator of Hormonal and Metabolic Responses toward *Botrytis cinerea* Infection . Plant Physiol. 2012;159: 266–285. doi:10.1104/pp.111.192641

82. Fountain JC, Raruang Y, Luo M, Brown RL, Guo B, Chen Z-Y. Potential roles of WRKY transcription factors in regulating host defense responses during Aspergillus flavus infection of immature maize kernels. Physiol Mol Plant Pathol. 2015;89: 31–40. doi:10.1016/j.pmpp.2014.11.005

83. Peng W, Li W, Song N, Tang Z, Liu J, Wang Y, et al. Genome-Wide Characterization, Evolution, and Expression Profile Analysis of GATA Transcription Factors in Brachypodium distachyon. Int J Mol Sci. 2021;22: 2026. doi:10.3390/ijms22042026

84. dos Santos C, Franco OL. Pathogenesis-Related Proteins (PRs) with Enzyme Activity Activating Plant Defense Responses. Plants. 2023;12: 2226. doi:10.3390/plants12112226

85. Feki K, Tounsi S, Jemli S, Boubakri H, Saidi MN, Mrabet M, et al. Genome-wide identification of PR10 family members and expression profile analysis of PvPR10 in common bean (*Phaseolus vulgaris* L.) in response to hormones and several abiotic stress conditions. Plant Growth Regul. 2023. doi:10.1007/s10725-023-00997-z

86. Song L, Wang J, Jia H, Kamran A, Qin Y, Liu Y, et al. Identification and functional characterization of NbMLP28, a novel MLP-like protein 28 enhancing Potato virus Y resistance in Nicotiana benthamiana. BMC Microbiol. 2020;20: 55. doi:10.1186/s12866-020-01725-7

87. Zhang J, Wang F, Liang F, Zhang Y, Ma L, Wang H, et al. Functional analysis of a pathogenesis-related thaumatin-like protein gene TaLr35PR5 from wheat induced by leaf rust fungus. BMC Plant Biol. 2018;18: 76. doi:10.1186/s12870-018-1297-2

88. Souza C de A, Li S, Lin AZ, Boutrot F, Grossmann G, Zipfel C, et al. Cellulose-Derived Oligomers Act as Damage-Associated Molecular Patterns and Trigger Defense-Like Responses. Plant Physiol. 2017;173: 2383–2398. doi:10.1104/pp.16.01680

89. Matiello RR, Barbieri RL, Carvalho FIF de. Resistência das plantas a moléstias fúngicas. Ciência Rural. 1997;27: 161–168. doi:10.1590/S0103-84781997000100028

90. Rojas CM, Senthil-Kumar M, Tzin V, Mysore KS. Regulation of primary plant metabolism during plant-pathogen interactions and its contribution to plant defense. Front Plant Sci. 2014;5. doi:10.3389/fpls.2014.00017

91. Chen Z, Kloek AP, Boch J, Katagiri F, Kunkel BN. The *Pseudomonas syringae avrRpt2* Gene Product Promotes Pathogen Virulence from Inside Plant Cells. Molecular Plant-Microbe Interactions®. 2000;13: 1312–1321. doi:10.1094/MPMI.2000.13.12.1312

92. Denancé N, Ranocha P, Oria N, Barlet X, Rivière M, Yadeta KA, et al. Arabidopsis wat1 (walls are thin1)-mediated resistance to the bacterial vascular pathogen, Ralstonia solanacearum, is accompanied by cross-regulation of salicylic acid and tryptophan metabolism. The Plant Journal. 2013;73: 225–239. doi:10.1111/tpj.12027

93. Ding X, Cao Y, Huang L, Zhao J, Xu C, Li X, et al. Activation of the Indole-3-Acetic Acid–Amido Synthetase GH3-8 Suppresses Expansin Expression and Promotes Salicylate- and Jasmonate-Independent Basal Immunity in Rice. Plant Cell. 2008;20: 228–240. doi:10.1105/tpc.107.055657

94. Kunkel BN, Harper CP. The roles of auxin during interactions between bacterial plant pathogens and their hosts. J Exp Bot. 2018;69: 245–254. doi:10.1093/jxb/erx447

95. O'Donnell PJ, Schmelz EA, Moussatche P, Lund ST, Jones JB, Klee HJ. Susceptible to intolerance – a range of hormonal actions in a susceptible *Arabidopsis* pathogen response. The Plant Journal. 2003;33: 245–257. doi:10.1046/j.1365-313X.2003.01619.x

96. Yin C, Park J-J, Gang DR, Hulbert SH. Characterization of a Tryptophan 2-Monooxygenase Gene from *Puccinia graminis* f. sp. *tritici* Involved in Auxin Biosynthesis and Rust Pathogenicity. Molecular Plant-Microbe Interactions®. 2014;27: 227–235. doi:10.1094/MPMI-09-13-0289-FI

97. Paxton JD. Phytoalexins — A Working Redefinition. Journal of Phytopathology. 1981;101: 106–109. doi:10.1111/j.1439-0434.1981.tb03327.x

98. Piasecka A, Jedrzejczak-Rey N, Bednarek P. Secondary metabolites in plant innate immunity: conserved function of divergent chemicals. New Phytologist. 2015;206: 948–964. doi:10.1111/nph.13325

99. Poppenberger B, Berthiller F, Lucyshyn D, Sieberer T, Schuhmacher R, Krska R, et al. Detoxification of the Fusarium Mycotoxin Deoxynivalenol by a UDP-glucosyltransferase from Arabidopsis thaliana. Journal of Biological Chemistry. 2003;278: 47905–47914. doi:10.1074/jbc.M307552200

100. Seifi HS, Van Bockhaven J, Angenon G, Höfte M. Glutamate Metabolism in Plant Disease and Defense: Friend or Foe? Molecular Plant-Microbe Interactions®. 2013;26: 475–485. doi:10.1094/MPMI-07-12-0176-CR

101. González-Mendoza VM, Sánchez-Sandoval ME, Castro-Concha LA, Hernández-Sotomayor SMT. Phospholipases C and D and Their Role in Biotic and Abiotic Stresses. Plants. 2021;10: 921. doi:10.3390/plants10050921

102. Krčková Z, Kocourková D, Daněk M, Brouzdová J, Pejchar P, Janda M, et al. The Arabidopsis thaliana non-specific phospholipase C2 is involved in the response to Pseudomonas syringae attack. Ann Bot. 2018;121: 297–310. doi:10.1093/aob/mcx160

103. Yang H, Wang D, Guo L, Pan H, Yvon R, Garman S, et al. Malectin/Malectin-like domain-containing proteins: A repertoire of cell surface molecules with broad functional potential. The Cell Surface. 2021;7: 100056. doi:10.1016/j.tcsw.2021.100056

Supporting information

S1 Fig. Expression profiles of genes differentially expressed by the cultivars Ouro Vermelho and Estilo, at 0, 48, and 96 hours after inoculation.

S1 Table. Genes, primers, and probes for qPCR assay.

S2 Table. Number of RNA sequencing reads for samples of the cultivars BRS Estilo and Ouro Vermelho at different hours after inoculation (hai) with the isolate Lv 134 of race 65 *Colletotrichum lindemuthianum* (0 hai, 48 hai, and 98 hai). The genomic reference used for sequencing alignment was *Phaseolus vulgaris* v2.1.

S3 Table. Differential expression analysis results for the different comparisons performed.

S4 Table. R48-like genes, S48-like genes, R96-like genes, and S96-like genes.

S9 Table. Gene Ontology (GO) enrichment terms of differentially expressed putative R and putative S genes in the different contrasts considering the times 0, 48, and 96 after inoculation of the Ouro Vermelho (resistant) and BRS Estilo (susceptible) common bean cultivars with the Lv 134 isolate of race 65 of Colletotrichum lindemuthianum.

S10 Table. Metabolic pathways associated with the putative resistance and susceptibility genes identified from different contrasts involving the BRS Estilo and Ouro Vermelho cultivars inoculated with the Lv 134 isolate of race 65 *Colletotrichum lindemuthianum*, at 0, 48 e 96 hours after innoculation.

S11 Table. Degree centrality measures for the gene coexpression network modeled for the Ouro Vermelho cultivar, highlighting the putative resistance genes.

S12 Table. Degree centrality measures for the gene coexpression network modeled for the BRS Estilo cultivar, highlighting the putative resistance genes.

S13 Table. Degree centrality measures for the gene coexpression network modeled for the Ouro Vermelho cultivar, highlighting the putative susceptibility genes.

S14 Table. Degree centrality measures for the gene coexpression network modeled for the BRS Estilo cultivar, highlighting the putative susceptibility genes.

Data Review URL

Click here to download Data Review URL

https://drive.google.com/drive/u/0/folders/16sdGfG8isTMyapbWTPb_XB
