# Supplementary material for: Comparative transcriptome analysis provides new insights into the response of common bean to infection by race 65 of Colletotrichum lindemuthianum
Source: PLoS One. 2025 Oct 17;20(10):e0314188. doi: 10.1371/journal.pone.0314188 (PMC12533873; doi:10.1371/journal.pone.0314188)
Supplement: Attachment — Submitted filename: Response to Reviewers.docx [file pone.0314188.s018.docx]

**Response to Reviewers [PONE-D-24-50843]**

We sincerely appreciate the reviewers’ valuable contributions, which have helped improve the quality of our manuscript. Below, we provide detailed responses to the comments.

**3. Have the authors made all data underlying the findings in their manuscript fully available?**

Reviewer #1: No

**Response:** We apologize for the inconvenience. All figures have now been incorporated and included at the end of the PDF. Additionally, the raw transcriptome data are being deposited in the NCBI database (BioProject ID: PRJNA1219552), ensuring full transparency and availability.

**4. Is the manuscript presented in an intelligible fashion and written in standard English?**

Reviewer #2: No

**Response:** The manuscript has undergone a thorough English revision to enhance clarity and precision.

**5. Review Comments to the Author**

Reviewer #1:

1) Bean anthracnose race 65 and its biotypes are widespread in Brazil. Although the introduction is fine. It would have been better to tell a reader how important race 65 and its biotypes are for Brazil.

**Response:** We have added information to the introduction to better contextualize its relevance. (L59-72)

2) L116 to L118. Have the dominant alleles Ouro Vermelho known? If yes please describe those alleles. A few recessive alleles also impart bean anthracnose resistance like co-6 and co-Indb. So, remodify the statement.

**Response:** We have revised the section to include the requested information on dominant and recessive alleles associated with anthracnose resistance. (L51-54)

3) There is a mention of susceptibility genes in GO analysis. Susceptibility genes are those which can be edited to make plants resistant. Instead of using the word susceptibility please remodify it to the appropriate word. This may confuse readers.

**Response:** We appreciate the observation. We have revised the entire text to ensure terminological accuracy and avoid potential ambiguities. We refer to the differentially expressed genes as ‘putative resistance genes’ (putative R48-genes or putative R96-genes), ‘putative susceptibility genes’ (putative S48-genes or putative S96-genes), ‘resistance-like genes’ (R48-like genes; R96-like genes), and ‘susceptibility-like genes’ (S48-like genes; S96-like genes), as described in the section "Differential expression analysis and enrichment of Gene Ontology terms" (L163-170). We believe these genes can only be definitively classified as resistance or susceptibility after further studies confirm their specific functions.

4) I was unable to find Figs mentioned in the text. So, I can’t comment on those. Authors should provide Figs with the manuscript. Because of these reasons, I am unable to move ahead with the manuscript. I am attaching a pdf that I reviewed. You will see that this pdf is without Figs and main Tables

**Response**: We have incorporated all figures and tables.

Minor Comments

**L92.** More studies are on the interaction of beans with race 73. Please check literature

**Response**: We reviewed the literature and added relevant information. (L89-101)

**L93** (2021) should be replaced with the correct number [--] (L96)

**Response**: References have been corrected as requested.

L94 (2016) should be replaced with the correct number [--] (L95; L93)

**Response**: References have been corrected as requested.

**Reviewer #2:** The manuscript has merit but is too long and needs modification in material and methods, results and discussion. It is necessary became short this topics. Many information should be in Supplemental information.

**Response**: We appreciate the feedback. We conducted a thorough and in-depth review of the entire text, from the Abstract to the Discussion, to make it more concise while ensuring that no content was compromised. The original version contained 8,367 words (including Table 1 and the legends for Figures 1-6). After the adjustments, the manuscript was reduced to 5,985 words, representing an approximate 30% reduction. We believe we have achieved a more direct and objective main text.

**Final Consideration:**

We thank the reviewers once again for their time spent reviewing our manuscript and for their suggestions. We hope the revisions and improvements meet their expectations. We are available for any further clarifications and look forward to the next stage of the evaluation process.

Best regards,

Welison Andrade Pereira
