# Supplementary material for: Comparative transcriptome analysis provides new insights into the response of common bean to infection by race 65 of Colletotrichum lindemuthianum
Source: PLoS One. 2025 Oct 17;20(10):e0314188. doi: 10.1371/journal.pone.0314188 (PMC12533873; doi:10.1371/journal.pone.0314188)
Supplement: Attachment — Submitted filename: Response to Reviewers [PONE-D-24-50843]_version2.docx [file pone.0314188.s019.docx]

**Response to Reviewers [PONE-D-24-50843]**

**Dear Dr. Karthikeyan Adhimoolam and Reviewers,**

We sincerely thank you for the insightful comments and constructive suggestions, which have substantially improved the clarity and scientific rigor of our manuscript. We have carefully addressed each point, and the corresponding revisions are highlighted in the tracked version of the manuscript for ease of review.

**Reviewer Comment 1:** ***Phaseolus vulgaris* is often written in full and sometimes as *P. vulgaris*. The same applies to *Colletotrichum lindemuthianum*. Authors can write their full names once and then refer to them as *P. vulgaris* and *C. lindemuthianum*.**

Response: We appreciate this important point regarding scientific nomenclature. We have standardized the usage of species names throughout the manuscript. The full scientific names *Phaseolus vulgaris* and *Colletotrichum lindemuthianum* were introduced upon their first mention in the Abstract and Introduction. Subsequently, their abbreviated forms (*P. vulgaris* and *C. lindemuthianum*) were consistently used throughout the remainder of the manuscript, except in section titles, figure captions, and table titles

**Reviewer Comment 2: The gene models listed in the text must be italicized.**

Response: We thank the reviewer for this correction. We have carefully reviewed the entire manuscript and italicized all gene models as per scientific convention.

**Reviewer Comment 3: L354. The genes must be italicized.**

Response: Thank you for your correction. We have now corrected this issue and have ensured that all gene names are properly italicized according to standard scientific practice.

**Reviewer Comment 4: L373. [59] please add author “xyz [59]”.**

Response: This context has been revised in the “Discussion” section to ensure accuracy, and the citation has been corrected accordingly.

**Reviewer Comment 5: L376. The pathogen is biotrophic for 72 hours and then switches to necrotrophic thereafter. Please refer to Mahiya-Farooq (2019) and Padder et al. (2016) for clarification.**

Response: We thank the reviewer for pointing out this inaccuracy regarding the pathogen's life cycle description. We have revised the statement to correctly reflect the duration of the biotrophic phase, as supported by the references provided.

**Changes implemented:**

- **Lines 383–388 now state:** “This is followed by infection vesicle formation and primary hyphae differentiation, marking the biotrophic phase, which lasts for approximately 72 hours, with the host cell remaining alive [60]. The fungus then transitions to the necrotrophic phase, characterized by secondary hyphae and host cell death [25,61,62]. Macroscopic symptoms appear at 96 hai [60,63], which was confirmed in the susceptible cultivar (BRS Estilo) in this study.”

**Reference added:**

62. Mahiya-Farooq, Padder BA, Bhat NN, Shah MD, Shikari AB, Awale HE, et al. Temporal expression of candidate genes at the *Co-1* locus and their interaction with other defense-related genes in common bean. *Physiol Mol Plant Pathol.* 2019;108: 101424. doi:10.1016/j.pmpp.2019.101424

**Reviewer Comment 6: Most of the discussion revolves around the repetition of results. The authors are encouraged to provide a more thorough discussion of these results.**

Response: We acknowledge the reviewer's valuable feedback on the discussion section. We have significantly revised this section to provide a more in-depth interpretation and contextualization of our results, moving beyond mere repetition. We focused on elaborating the implications of our findings, connecting them more explicitly to existing literature and broader biological understanding of plant-pathogen interactions.

**Reviewer Comment 7:** **L451, L470, L534. Italicize the words *Arabidopsis* and *Sclerotinia*.**

**Response:** We have revised the manuscript to italicize all occurrences of the scientific names Arabidopsis and Sclerotinia, in accordance with nomenclature standards.

**Reviewer Comment 8: Authors must check the references. All scientific names in the title should be italicized. Please double-check each cited reference.**

Response: We conducted a careful review of all references, using the Mendeley tool to support the revisions. Subsequently, each entry in the list was checked for compliance with the journal’s formatting guidelines. In particular, all scientific names, including taxonomic genera, appearing in the titles of the cited articles have been properly italicized.

On behalf of all co-authors, we sincerely thank the reviewers and the editorial team for their constructive feedback. We believe that the revisions have considerably improved the clarity, accuracy, and scientific value of the manuscript, and we look forward to its reconsideration. We remain at the reviewers’ and editorial team’s disposal for any further clarifications.

Sincerely,
On behalf of all co-authors,

Prof. Welison Andrade Pereira

Federal University of Lavras (UFLA), Brazil
